# Rapid and One-Pot Synthesis of Aryl Ynamides from Aryl Alkynyl Acids by Metal-Free C-N Cleavage of Tertiary Amines

**DOI:** 10.3390/molecules30142955

**Published:** 2025-07-13

**Authors:** Yong Liu, Xiaoyong Liu, Hongwei Li, Shengmei Guo

**Affiliations:** 1 Ganzhou Institute for Cancer Research, Ganzhou 341000, China; 182791347852@163.com; 2 Department of Chemistry, Nanchang University, Nanchang 330031, China; 402800220041@email.ncu.edu.cn

**Keywords:** aryl ynamide, C-N bond cleavage, tertiary amine, one-pot synthesis

## Abstract

Herein a rapid, metal-free, and highly efficient synthesis of aryl ynamides from aryl alkynyl acids has been described. This approach, utilizing tertiary amines as an amino source via metal-free C-N cleavage, enabled the construction of a diverse range of aryl ynamides with medium to excellent yields (33 examples, up to 95% yield). This reaction exhibits significantly enhanced efficiency compared to the conventional stepwise approach involving aryl alkynyl acids and secondary amines. It can be successfully scaled up, providing a practical and environmentally benign strategy for alkynamide synthesis.

## 1. Introduction

**Scheme 1:**
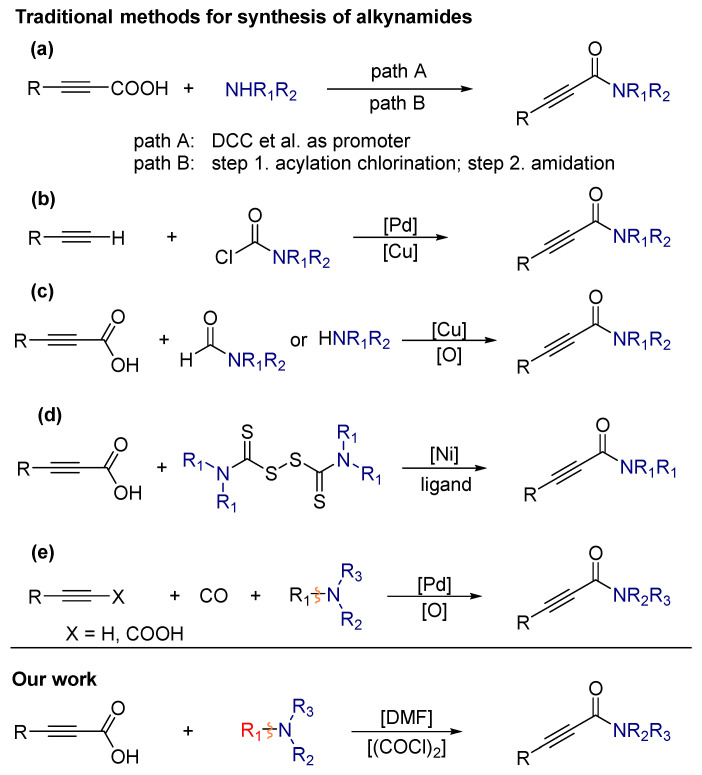
Synthesis of ynamides.

Ynamides are pivotal structural motifs in natural and pharmaceutical molecules [1,2,3]. They are also recognized as versatile intermediates in heterocyclic synthesis, making them a focal point of extensive research in organic synthesis [4,5,6,7,8]. Traditionally, ynamides are synthesized via amidation of alkynyl acids and amines in the presence of DCC and DMAP or by two steps (Scheme 1a) [9,10,11,12,13]: (1) aryl propiolic acids are first converted into chlorides via reaction with oxalyl chloride or thionyl chloride; (2) Subsequently, the chlorides are treated with primary or secondary amines in the presence of a base to afford the corresponding products. Though efficient, this method produces a large amount of waste, lacks step economy, and needs long reaction times. Recently, the Pd/Cu-catalyzed cross-coupling reaction between alkynes and amineformyl chloride successfully realized the preparation of these ynamides (Scheme 1b) [14,15,16]. The Cu/TBHP-mediated synthesis of these compounds was also accomplished between secondary amines or formamides with alkynyl acids (Scheme 1c) [17,18,19,20,21,22]. Nevertheless, these approaches are plagued by issues such as multi-step synthesis procedures and poor stability of amine formyl chloride [23]. Further progress was achieved by Shi and co-workers, who demonstrated a Ni-catalyzed coupling between alkynyl acids and tetraalkylthiuram disulfides under ligand-free conditions (Scheme 1d) [24].

Tertiary amines have garnered significant interest in organic synthesis due to their exceptional stability and ready availability [25,26,27,28,29,30]. Since 2010 and the pioneering work by Huang on the selective C-N bond cleavage of tertiary amines by transition-metal catalysis [31], tremendous progress has been achieved in the development of powerful methods for the introduction of amino groups into molecules [32,33,34]. This strategy has also been employed in the ynamide synthesis. The Bhanage and Lee groups independently developed Pd-catalyzed N-dealkylation/carbonylation protocols for the synthesis of ynamides (Scheme 1e) [35,36]. While these methods depend on noble metal catalysts, they face challenges in terms of sustainability, cost-effectiveness, and operational simplicity [37]. To address this unmet need, we herein report a rapid, one-pot synthesis of aryl ynamides of alkynyl acids employing inexpensive tertiary amines as amino sources under catalyst-free conditions [38,39,40,41,42,43,44].

## 2. Results and Discussion

We chose phenylpropynoic acid (**1a**, 0.2 mmol) and triethylamine (**2a**, 0.7 mmol) as the model substrates (Table 1). Initially, DMF (0.6 equiv.) and (COCl)_2_ (1.5 equiv.) were employed as additives with DCM (0.5 mL) serving as the solvent. The reaction was conducted at room temperature for 2.0 h under a nitrogen atmosphere. Ultimately, the target product **3a** (Table 1, entry 1) was successfully obtained in an excellent yield of 94%. Subsequently, the effect of the solvent on the reaction was screened. When MeCN, toluene, and dioxane were utilized as the solvents, only moderate to low yields were achieved (Table 1, entries 2–4). Additionally, when DMSO and MeOH were employed as solvents, there was no product detected in this reaction (Table 1, entries 5 and 6). Interestingly, DMF was found to promote the reaction efficiency significantly. Control experiments revealed that the reaction proceeded with only 66% yield in the absence of DMF, demonstrating its crucial role as a reaction accelerator (Table 1, entry 7). While increasing the loading of DMF to 0.8 eq., the yield of **3a** was increased to 93% (Table 1, entries 8–10). Next, the reaction time was investigated. When the reaction time was shortened to 0.5 h, only 47% of **3a** was produced. Further extending the reaction time to 1.0 h, 1.5 h, and 2.0 h, the desired products were obtained in progressively higher yields of 75%, 86%, and 94%, respectively (Table 1, entries 11–14).

**Scheme 2:**
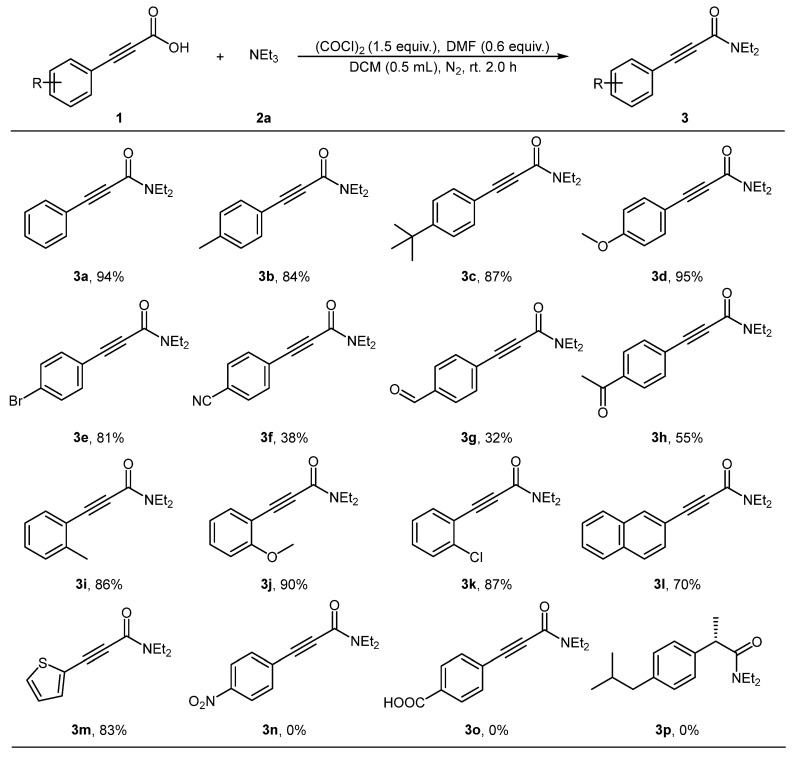
Scope of propionic acids. Reaction condition: **1** (0.2 mmol), **2a** (3.5 equiv.), (COCl)_2_ (1.5 equiv.), DMF (0.6 equiv.), DCM (0.5 mL), rt, under nitrogen atmosphere for 2.0 h; Isolated yields.

After the optimal reaction conditions were determined, the substrate compatibility study on the 3-arylpropynoic acids was conducted (Scheme 2). First, the substrates with electron-rich substituent groups such as -CH_3_ (**3b**), -C(CH_3_)_3_ (**3c**), and -OCH_3_ (**3d**) at the para-position of the arene showed good reactivity, and the corresponding alkynamides could be obtained in good yields. In addition, halogen substituents on the benzene ring were also compatible with this reaction. For example, 3-(4-bromophenyl)propiolic acid delivered **3e** in 81%. Electron-deficient groups such as -CN, -CHO, and -COCH_3_ were introduced to the aryl para-position, and it was found that the conversion was inhibited, with only moderate yields (**3f**–**3h**). Ortho-substituted arylpropylic acids were investigated, and it was found that there were no effects of steric hindrance on the reactions, with 86–90% yields, respectively (**3i**–**3k**). It is gratifying that except for phenyl, naphthyl (**3l**)- and heteroaryl (**3m**)-substituted arylpropylic acids were also compatible with the transformation, furnishing the target amides in 70% and 83% yields, respectively. Unfortunately, the substrates with -NO_2_ (**3n**) and -COOH (**3o**) groups did not participate in the reaction. Finally, ibuprofen (**3p**) was used as a partner with triethylamine under the standard conditions, but no product was produced.

**Scheme 3:**
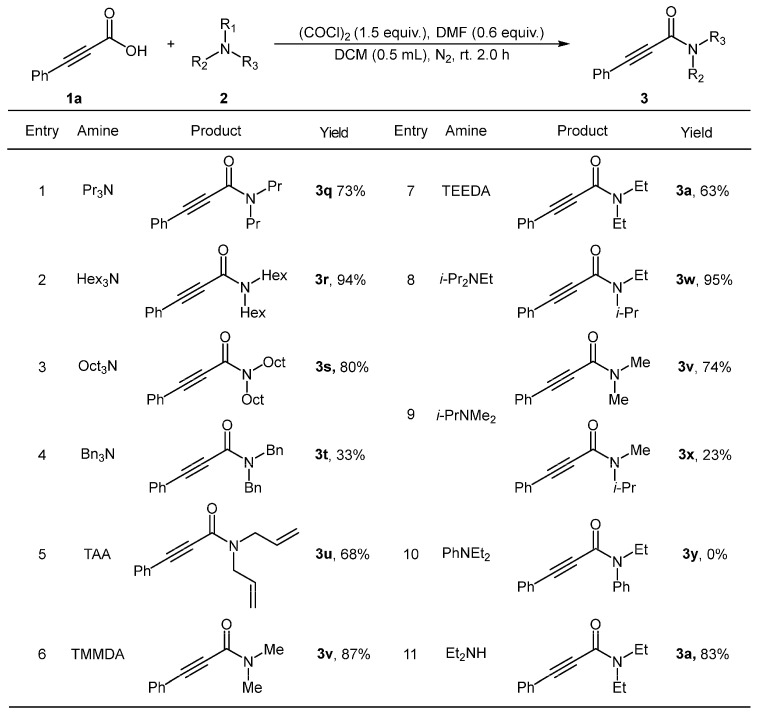
Scope of propionic acids. Reaction condition: **1a** (0.2 mmol), **2** (3.5 equiv.), (COCl)_2_ (1.5 equiv.), DMF (0.6 equiv.), DCM (0.5 mL), rt, under nitrogen atmosphere for 2.0 h; Isolated yields. TAA = triallylamine; TMMDA = *N,N,N’,N’*-tetramethylmethanediamine; TEEDA = *N*^1^,*N*^1^,*N*^2^,*N*^2^-tetraethylethane-1,2-diamine.

Next, the scope of tertiary amines was expanded (Scheme 3). For symmetric alkyl tertiary amines such as Pr_3_N, Hex_3_N, and Oct_3_N, the reactions could be carried out smoothly, giving the desired products **3q**–**3s** in good yields. The yield was drastically reduced when Bn_3_N was employed in the reactions (**3t**), which may be due to the large steric hindrance, making the reaction difficult. It is gratifying that the unsaturated triallylamine showed high compatibility, which achieved the corresponding product in 68% yield (**3u**). Subsequently, we turned our attention to asymmetric tertiary amines. TMMDA, TEEDA, and DIPEA—each containing two distinct C-N bond cleavage sites—demonstrated remarkable regioselectivity in their reactions; only a single product was detected under these reaction conditions (**3v**, **3a**, and **3w**). However, employing *N,N*-dimethylisopropylamine as the substrate led to the formation of two N-dealkylation/carbonylation products (**3v** and **3x**) in an approximate 3:1 ratio. Unfortunately, we did not observe the amidation product when PhNEt_2_ was used as a tertiary amine source (**3y**). The secondary amine Et_2_NH was also added in the reaction, and the result showed inferior to Et_3_N (**3a**) under the same conditions, suggesting that this work provides an advantage over traditional methods.

**Scheme 4:**
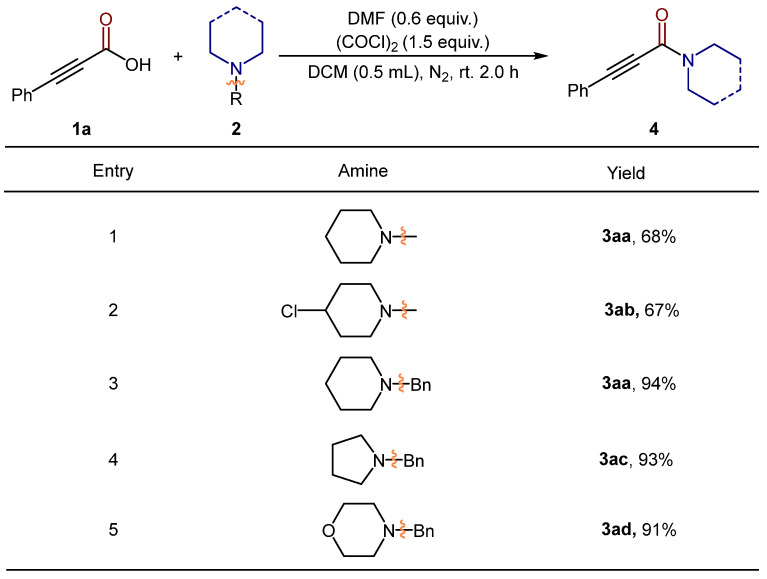
Scope of propynoic acids. Reaction condition: **1a** (0.2 mmol), **2** (3.5 equiv.), (COCl)_2_ (1.5 equiv.), DMF (0.6 equiv.), DCM (0.5 mL), rt, under nitrogen atmosphere for 2.0 h; Isolated yields.

Finally, the cyclic tertiary amines were also explored (Scheme 4). Our findings revealed that the majority of N-methyl cyclic tertiary amines underwent smooth conversion processes, offering the demethylation products in moderate yields (**3aa**, **3ab**). Additionally, owing to the conjugation effect exerted by the benzyl group, the benzyl moiety proved to be a more favorable leaving group than the methyl group. Consequently, N-benzyl-substituted cyclic tertiary amines demonstrated notably higher yields (**3ac**, **3ad**).

**Scheme 5:**
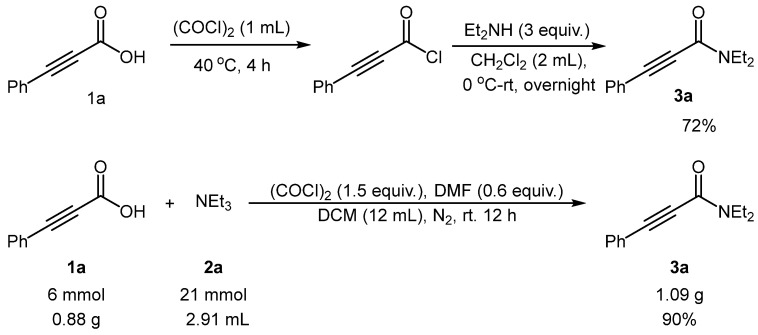
Scope of propionic acids. Reaction condition: **1** (0.2 mmol), **2a** (3.5 equiv.), (COCl)_2_ (1.5 equiv.), DMF (0.6 equiv.), DCM (0.5 mL), rt, under nitrogen atmosphere for 2.0 h; Isolated yields.

To quantify the improvements of our one-pot synthetic method relative to conventional methods, we performed control experiments using the conventional two-step approach with phenylpropynoic acid and Et_2_NH, which afforded the desired product in 72% yield. To further evaluate the scalability and practical applicability of this strategy, a gram-scale reaction was successfully conducted with a yield of 90% (Scheme 5).

## 3. Materials and Methods

### 3.1. General Information

All commercially available reagent-grade chemicals were purchased from Adamas (Shanghai, China), Aldrich (St. Louis, MO, USA), Accela (San Ramon, CA, USA), Alfa Aesar (Ward Hill, MA, USA), and TCI (Portland, OR, USA) and were used as received without further purification unless otherwise stated. ^1^H NMR, ^13^C NMR were recorded in CDCl_3_ on a Bruker Avance III 400 spectrometer with TMS as internal standard (400 MHz ^1^H, 101 MHz ^13^C, Bruker, Billerica, MA, USA) at room temperature; the chemical shifts (δ) were expressed in ppm, and J values were given in Hz. HRMS was performed by the Analysis and Testing Center, Nanchang University. The following abbreviations are used to indicate the multiplicity: singlet (s), doublet (d), triplet (t), quartet (q), doublet of doublets (dd), doublet of triplets (dt), and multiplet (m). All first-order splitting patterns were assigned based on the appearance of the multiplet. Splitting patterns that could not be easily interpreted were designated as multiplet (m). Column chromatography was performed on silica gel (200–300 mesh).

### 3.2. Experimental Procedure for Ynamides

To a dry round-bottom flask, aryl alkynyl acids, oxalyl chloride, DMF, tertiary amines, and CH_2_Cl_2_ were added. The mixture was stirred under an N_2_ atmosphere for 2 h at room temperature. After the reaction was completed, the solvent was removed by rotary evaporation, and the residual solution was extracted with CH_2_Cl_2_ (3 × 15 mL). Then, the organic layer was dried with anhydrous Na_2_SO_4_. Finally, after filtration, the solvent was evaporated under reduced pressure. The crude product was purified by silica gel column chromatography to obtain the target compounds.

*N,N-Diethyl-3-phenylpropiolamide* (**3a**): Yellow liquid and the yield is 94%; ^1^H NMR (400 MHz, CDCl_3_) δ 7.50–7.45 (m, 2H), 7.38–7.27 (m, 3H), 3.61 (q, *J* = 7.1 Hz, 2H), 3.42 (q, *J* = 7.1 Hz, 2H), 1.22 (t, *J* = 7.1 Hz, 3H), 1.12 (t, *J* = 7.2 Hz, 3H). ^13^C NMR (101 MHz, CDCl_3_) δ 153.71, 132.05, 129.66, 128.27, 120.49, 88.71, 81.73, 43.39, 39.10, 14.18, 12.64. HRMS: calcd for C_13_H_16_ON [M + H]^+^: 202.1226, found: 202.1222.

*N,N-Diethyl-3-(p-tolyl)propiolamide* (**3b**): Yellow liquid and the yield is 84%; ^1^H NMR (400 MHz, CDCl_3_) δ 7.42 (d, *J* = 8.3 Hz, 2H), 7.15 (d, *J* = 7.8 Hz, 2H), 3.69–3.61 (m, 2H), 3.46 (q, *J* = 7.1 Hz, 2H), 2.36 (s, 3H), 1.27 (t, *J* = 7.1 Hz, 3H), 1.16 (t, *J* = 7.1 Hz, 3H). ^13^C NMR (101 MHz, CDCl_3_) δ 154.09, 140.29, 132.22, 129.20, 117.57, 89.34, 81.44, 43.54, 39.22, 21.58, 14.34, 12.83. HRMS: calcd for C_14_H_18_ON [M + H]^+^: 216.1383, found: 216.1329.

*3-(4-(Tert-butyl)phenyl)-N,N-diethylpropiolamide* (**3c**): Yellow liquid and the yield is 87%; ^1^H NMR (400 MHz, CDCl_3_) δ 7.46 (d, *J* = 8.3 Hz, 2H), 7.37 (d, *J* = 8.5 Hz, 2H), 3.65 (q, *J* = 7.1, 6.6 Hz, 2H), 3.46 (q, *J* = 7.1 Hz, 2H), 1.30 (s, 9H), 1.26 (t, *J* = 7.0 Hz, 3H), 1.17 (t, *J* = 7.1 Hz, 3H). ^13^C NMR (101 MHz, CDCl_3_) δ 154.05, 153.31, 132.05, 125.43, 117.58, 89.24, 81.41, 43.52, 39.22, 34.84, 30.97, 14.30, 12.79. HRMS: calcd for C_17_H_24_ON [M + H]^+^: 258.1852, found: 258.1845.

*N,N-Diethyl-3-(4-methoxyphenyl)propiolamide* (**3d**): Yellow liquid and the yield is 95%; ^1^H NMR (400 MHz, CDCl_3_) δ 7.47 (d, *J* = 7.0 Hz, 2H), 6.86 (d, *J* = 6.9 Hz, 2H), 3.81 (s, 3H), 3.64 (q, *J* = 7.2 Hz, 2H), 3.45 (q, *J* = 7.1 Hz, 2H), 1.26 (t, *J* = 7.1 Hz, 3H), 1.16 (t, *J* = 8.0 Hz, 3H). ^13^C NMR (101 MHz, CDCl_3_) δ 160.82, 154.23, 134.02, 114.12, 112.59, 89.46, 81.12, 55.30, 43.52, 39.20, 14.33, 12.84. HRMS: calcd for C_14_H_18_O_2_N [M + H]^+^: 232.1332, found: 232.1328.

*3-(4-Bromophenyl)-N,N-diethylpropiolamide* (**3e**): Yellow liquid and the yield is 81%; ^1^H NMR (400 MHz, CDCl_3_) δ 7.49 (d, *J* = 8.3 Hz, 2H), 7.37 (d, *J* = 8.3 Hz, 2H), 3.63 (q, *J* = 7.2 Hz, 2H), 3.46 (q, *J* = 7.2 Hz, 2H), 1.26 (t, *J* = 7.2 Hz, 3H), 1.16 (t, *J* = 7.2 Hz, 3H). ^13^C NMR (101 MHz, CDCl_3_) δ 153.64, 133.60, 131.79, 124.41, 119.59, 87.76, 82.79, 43.54, 39.28, 14.36, 12.77. HRMS: calcd for C_13_H_15_BrON [M + H]^+^: 280.0332, found: 280.0329.

*3-(4-Cyanophenyl)-N,N-diethylpropiolamide* (**3f**): Yellow liquid and the yield is 38%; ^1^H NMR (400 MHz, CDCl_3_) δ 7.63 (q, *J* = 8.4, 7.9 Hz, 4H), 3.64 (q, *J* = 7.1 Hz, 2H), 3.47 (q, *J* = 7.0 Hz, 2H), 1.27 (t, *J* = 7.2 Hz, 3H), 1.18 (t, *J* = 7.2 Hz, 3H). ^13^C NMR (101 MHz, CDCl_3_) δ 153.11, 132.66, 132.12, 125.53, 117.95, 113.21, 86.53, 85.22, 43.58, 39.40, 14.39, 12.73. HRMS: calcd for C_14_H_15_ON_2_ [M + H]^+^: 227.1179, found: 227.1174.

*N,N-Diethyl-3-(4-formylphenyl)propiolamide* (**3g**): Yellow liquid and the yield is 32%; ^1^H NMR (400 MHz, CDCl_3_) δ 10.03 (s, 1H), 7.88 (d, *J* = 8.5 Hz, 2H), 7.68 (d, *J* = 8.2 Hz, 2H), 3.66 (q, *J* = 7.1 Hz, 2H), 3.48 (q, *J* = 7.2 Hz, 2H), 1.29 (t, *J* = 7.2 Hz, 3H), 1.18 (t, *J* = 7.2 Hz, 3H). ^13^C NMR (101 MHz, CDCl_3_) δ 191.20, 153.34, 136.49, 132.76, 129.52, 126.73, 87.43, 84.83, 43.59, 39.37, 14.42, 12.78. HRMS: calcd for C_14_H_16_O_2_N [M + H]^+^: 230.2870, found: 230.2867.

*3-(4-Acetylphenyl)-N,N-diethylpropiolamide* (**3h**): Yellow liquid and the yield is 55%; ^1^H NMR (400 MHz, CDCl_3_) δ 7.93 (d, *J* = 8.4 Hz, 2H), 7.60 (d, *J* = 8.5 Hz, 2H), 3.65 (q, *J* = 7.0 Hz, 2H), 3.47 (q, *J* = 7.1 Hz, 2H), 2.60 (s, 3H), 1.27 (t, *J* = 7.2 Hz, 3H), 1.17 (t, *J* = 7.2 Hz, 3H). ^13^C NMR (101 MHz, CDCl_3_) δ 197.05, 153.44, 137.40, 132.35, 128.21, 125.34, 87.65, 84.31, 43.58, 39.33, 26.60, 14.36, 12.74. HRMS: calcd for C_15_H_18_O_2_N [M + H]^+^: 244.1332, found: 244.1328.

*N,N-Diethyl-3-(o-tolyl)propiolamide* (**3i**): Yellow liquid and the yield is 86%; ^1^H NMR (400 MHz, CDCl_3_) δ 7.50 (d, *J* = 7.8 Hz, 1H), 7.28 (t, *J* = 7.5 Hz, 1H), 7.21 (d, *J* = 7.6 Hz, 1H), 7.16 (t, *J* = 7.5 Hz, 1H), 3.66 (q, *J* = 6.7 Hz, 2H), 3.47 (q, *J* = 7.1, 6.6 Hz, 2H), 2.46 (s, 3H), 1.26 (t, *J* = 7.2 Hz, 3H), 1.17 (t, *J* = 7.2 Hz, 3H). ^13^C NMR (101 MHz, CDCl_3_) δ 154.03, 141.06, 132.88, 129.81, 129.57, 125.68, 120.52, 87.98, 85.67, 43.52, 39.28, 20.58, 14.40, 12.81. HRMS: calcd for C_14_H_18_ON [M + H]^+^: 216.1383, found: 216.1328.

*N,N-Diethyl-3-(2-methoxyphenyl)propiolamide* (**3j**): Yellow liquid and the yield is 90%; ^1^H NMR (400 MHz, CDCl_3_) δ 7.48 (d, *J* = 7.6 Hz, 1H), 7.35 (t, *J* = 7.6 Hz, 1H), 6.91 (t, *J* = 7.6 Hz, 1H), 6.87 (d, *J* = 8.4 Hz, 1H), 3.85 (s, 3H), 3.70 (q, *J* = 7.1 Hz, 2H), 3.46 (q, *J* = 7.1 Hz, 2H), 1.26 (t, *J* = 7.1 Hz, 3H), 1.15 (t, *J* = 7.1 Hz, 3H). ^13^C NMR (101 MHz, CDCl_3_) δ 161.08, 154.17, 134.13, 131.41, 120.41, 110.57, 109.96, 86.00, 85.53, 55.62, 43.50, 39.17, 14.28, 12.83. HRMS: calcd for C_14_H_18_O_2_N [M + H]^+^: 232.1332, found: 232,1328.

*3-(2-Chlorophenyl)-N,N-diethylpropiolamide* (**3k**): Yellow liquid and the yield is 87%; ^1^H NMR (400 MHz, CDCl_3_) δ 7.59 (d, *J* = 7.4 Hz, 1H), 7.40 (t, *J* = 5.6 Hz, 1H), 7.32 (t, *J* = 7.4 Hz, 1H), 7.25 (d, *J* = 7.4 Hz, 1H), 3.71 (q, *J* = 7.0 Hz, 2H), 3.46 (q, *J* = 7.1 Hz, 2H), 1.26 (t, *J* = 7.1 Hz, 3H), 1.26 (t, *J* = 7.1 Hz, 3H). ^13^C NMR (101 MHz, CDCl_3_) δ 153.61, 136.67, 134.42, 130.89, 129.35, 126.65, 120.88, 86.44, 85.13, 43.56, 39.34, 14.45, 12.78. HRMS: calcd for C_13_H_15_ClON [M + H]^+^: 236.0837, found: 286.0834.

*N,N-Diethyl-3-(naphthalen-2-yl)propiolamide* (**3l**): Yellow liquid and the yield is 70%; ^1^H NMR (400 MHz, CDCl_3_) δ 8.08 (s, 1H), 7.81 (dd, *J* = 7.3, 4.4 Hz, 3H), 7.56–7.48 (m, 3H), 3.70 (q, *J* = 7.1 Hz, 2H), 3.49 (q, *J* = 7.2 Hz, 2H), 1.31 (t, *J* = 7.1 Hz, 3H), 1.19 (t, *J* = 7.2 Hz, 3H). ^13^C NMR (101 MHz, CDCl_3_) δ 153.94, 133.39, 132.99, 132.60, 128.20, 128.05, 127.92, 127.76, 127.46, 126.81, 117.87, 89.37, 82.06, 43.59, 39.27, 14.40, 12.82. HRMS: calcd for C_17_H_18_ON [M + H]^+^: 252.3370, found: 252.3368.

*N,N-Diethyl-3-(thiophen-2-yl)propiolamide* (**3m**): Yellow liquid and the yield is 83%; ^1^H NMR (400 MHz, CDCl_3_) δ 7.38 (t, *J* = 3.4 Hz, 2H), 7.02 (t, *J* = 4.3 Hz, 1H), 3.61 (q, *J* = 7.2 Hz, 2H), 3.45 (q, *J* = 7.2 Hz, 2H), 1.25 (t, *J* = 7.1 Hz, 3H), 1.15 (t, *J* = 7.2 Hz, 3H). ^13^C NMR (101 MHz, CDCl_3_) δ 153.70, 134.79, 129.72, 127.30, 120.36, 85.83, 82.69, 43.47, 39.21, 14.34, 12.79. HRMS: calcd for C_11_H_14_OSN [M + H]^+^: 208.2990, found: 208.2986.

*3-Phenyl-N,N-dipropylpropiolamide* (**3q**): Yellow liquid and the yield is 73%; ^1^H NMR (400 MHz, CDCl_3_) δ 7.52 (d, *J* = 7.3 Hz, 2H), 7.42–7.33 (m, 3H), 3.56 (t, *J* = 7.4 Hz, 2H), 3.36 (t, *J* = 7.4 Hz, 2H), 1.73–1.66 (m, 2H),1.63–1.56 (m, 2H), 0.96 (t, *J* = 7.4 Hz, 3H), 0.92 (t, *J* = 7.4 Hz, 3H). ^13^C NMR (101 MHz, CDCl_3_) δ 154.53, 132.25, 129.82, 128.46, 120.78, 89.26, 82.11, 50.84, 46.50, 22.17, 20.69, 11.32, 11.23. HRMS: calcd for C_15_H_20_ON [M + H]^+^: 230.1539, found: 230.1536.

*N,N-Dihexyl-3-phenylpropiolamide* (**3r**): Yellow liquid and the yield is 94%; ^1^H NMR (400 MHz, CDCl_3_) δ 7.52 (d, *J* = 7.0 Hz, 2H), 7.42–7.33 (m, 3H), 3.58 (t, *J* = 7.5 Hz, 2H), 3.38 (t, *J* = 7.5 Hz, 2H), 1.69–1.61 (m, 2H), 1.60–1.53 (m, 2H), 1.37–1.25 (m, 12H), 0.90–0.84 (m, 6H). ^13^C NMR (101 MHz, CDCl_3_) δ 154.33, 132.21, 129.76, 128.42, 120.79, 89.13, 82.14, 54.09, 49.11, 44.81, 31.78, 31.53, 28.82, 27.41, 26.59, 26.38, 22.51, 13.96, 13.90. HRMS: calcd for C_21_H_32_ON [M + H]^+^: 314.2478, found: 314.2474.

*N,N-Dioctyl-3-phenylpropiolamide* (**3s**): Yellow liquid and the yield is 80%; ^1^H NMR (400 MHz, CDCl_3_) δ 7.52 (d, *J* = 7.3 Hz, 2H), 7.41–7.41 (m, 3H), 3.58 (t, *J* = 7.5 Hz, 2H), 3.38 (t, *J* = 7.5 Hz, 2H), 1.65 (t, *J* = 7.3 Hz, 2H), 1.56 (t, *J* = 7.4 Hz, 2H), 1.34–1.23 (m, 20H), 0.89–0.83 (m, 6H). ^13^C NMR (101 MHz, CDCl_3_) δ 154.40, 132.26, 129.80, 128.46, 120.83, 89.22, 82.17, 53.91, 49.18, 44.87, 31.77, 31.74, 29.34, 29.26, 29.20, 28.89, 27.48, 26.98, 26.75, 22.61, 22.58, 14.05, 14.03. HRMS: calcd for C_25_H_40_ON [M + H]^+^: 370.3104, found: 370.3100.

*N,N-Dibenzyl-3-phenylpropiolamide* (**3t**): Yellow liquid and the yield is 33%; ^1^H NMR (400 MHz, CDCl_3_) δ 7.52 (s, 1H), 7.50 (s, 1H), 7.43–7.24 (m, 13H), 4.76 (s, 2H), 4.57 (s, 2H). ^13^C NMR (101 MHz, CDCl_3_) δ 154.98, 136.19, 136.03, 132.41, 130.09, 128.85, 128.66, 128.47, 128.43, 127.93, 127.69, 127.62, 120.31, 90.80, 81.60, 51.41, 46.34. HRMS: calcd for C_23_H_20_ON [M + H]^+^: 326.1539, found: 326.1534.

*N,N-Diallyl-3-phenylpropiolamide* (**3u**): Yellow liquid and the yield is 68%; ^1^H NMR (400 MHz, _CDCl3_) δ 7.53 (d, *J* = 7.2 Hz, 2H), 7.44–7.32 (m, 3H), 5.89–5.71 (m, 2H), 5.27–5.15 (m, 4H), 4.22 (d, *J* = 5.8 Hz, 2H), 4.06 (d, *J* = 6.0 Hz, 2H). ^13^C NMR (101 MHz, CDCl_3_) δ 154.41, 132.69, 132.33, 132.09, 129.99, 128.45, 120.43, 118.07, 117.94, 89.87, 81.47, 50.72, 46.37. HRMS: calcd for C_15_H_16_ON [M + H]^+^: 226.1226, found: 226.1221.

*N,N-Dimethyl-3-phenylpropiolamide* (**3v**): Yellow liquid and the yield is 87%; ^1^H NMR (400 MHz, CDCl_3_) δ 7.53 (d, *J* = 8.0 Hz, 2H), 7.43–7.32 (m, 3H), 3.28 (s, 3H), 3.02 (s, 3H). ^13^C NMR (101 MHz, CDCl_3_) δ 154.59, 132.28, 129.91, 128.45, 120.56, 90.12, 81.53, 38.34, 34.13. HRMS: calcd for C_11_H_12_ON [M + H]^+^: 174.0913, found: 174.0908.

*N-Ethyl-N-isopropyl-3-phenylpropiolamide* (**3w**): Yellow liquid and the yield is 95%; ^1^H NMR (400 MHz, CDCl_3_) δ 7.52 (d, *J* = 6.8 Hz, 2H), 7.53–7.32 (m, 3H), 4.74–4.65 (m, 1H), 3.55 (q, *J* = 7.2 Hz, 1H), 3.34 (q, *J* = 7.0 Hz, 1H), 1.32 (t, *J* = 7.4 Hz, 1H), 1.25 (d, *J* = 7.3 Hz, 4H), 1.19 (d, *J* = 7.2 Hz, 4H). ^13^C NMR (101 MHz, CDCl_3_) δ 154.35, 153.85, 132.20, 132.18, 129.75, 129.70, 128.40, 128.37, 89.35, 88.53, 82.54, 81.91, 50.52, 45.33, 39.22, 35.36, 21.21, 20.30, 16.58, 14.45. HRMS: calcd for C_14_H_18_ON [M + H]^+^: 216.1383, found: 216.1379.

*N-Isopropyl-N-methyl-3-phenylpropiolamide* (**3x**): Yellow liquid and the yield is 23%; ^1^H NMR (400 MHz, CDCl_3_) δ 7.53 (d, *J* = 6.7 Hz, 2H), 7.42–7.33 (m,3H), 4.89–4.71 (m, 1H), 3.12 (s, 1H), 2.86 (s, 2H), 1.24 (d, *J* = 6.7 Hz, 4H), 1.15 (d, *J* = 6.8 Hz, 2H). ^13^C NMR (101 MHz, CDCl_3_) δ 154.31, 154.09, 132.28, 129.83, 128.44, 120.72, 90.03, 89.95, 82.16, 81.50, 50.09, 43.96, 29.79, 25.57, 20.36, 19.22. HRMS: calcd for C_13_H_16_ON [M + H]^+^: 202.1226, found: 202.1222.

*3-Phenyl-1-(piperidin-1-yl)prop-2-yn-1-one* (**3aa**): Yellow liquid and the yield is 68%; ^1^H NMR (400 MHz, CDCl_3_) δ 7.54 (d, *J* = 7.4 Hz, 2H), 7.42–7.33 (m, 3H), 3.77 (t, *J* = 5.1 Hz, 2H), 3.62 (t, *J* = 5.5 Hz, 2H), 1.66 (t, *J* = 7.4 Hz, 4H), 1.61–1.55 (m, 2H). ^13^C NMR (101 MHz, CDCl_3_) δ 152.89, 132.26, 129.81, 128.42, 120.68, 90.20, 81.41, 48.17, 42.32, 26.40, 25.34, 24.48. HRMS: calcd for C_14_H_16_ON [M + H]^+^: 214.1226, found: 214.1223.

*1-(4-Chloropiperidin-1-yl)-3-phenylprop-2-yn-1-one* (**3ab**): Yellow liquid and the yield is 67%; ^1^H NMR (400 MHz, CDCl_3_)δ7.56–7.51 (m, 2H), 7.45–7.33 (m, 3H), 4.36–4.31 (m, 1H), 4.07–3.98 (m, 1H), 3.88–3.79 (m, 2H), 3.77–3.69 (m, 1H), 2.18–2.02 (m, 2H), 1.99–1.83 (m, 2H). ^13^C NMR (101 MHz, CDCl_3_) δ 152.94, 132.32, 130.07, 128.49, 120.32, 90.81, 80.89, 56.17, 44.01, 38.23, 35.12, 34.14. HRMS: calcd for C_14_H_15_ClON [M + H]^+^: 248.0837, found: 248.0836.

*3-Phenyl-1-(pyrrolidin-1-yl)prop-2-yn-1-one* (**3ac**): Yellow liquid and the yield is 93%; ^1^H NMR (400 MHz, CDCl_3_) δ 7.52 (d, *J* = 8.3 Hz, 2H), 7.41–7.32 (m, 3H), 3.71 (t, *J* = 6.5 Hz, 2H), 3.51 (t, *J* = 6.4 Hz, 2H), 1.99–1.90 (m, 4H). ^13^C NMR (101 MHz, CDCl_3_) δ 152.65, 132.31, 129.85, 128.41, 120.56, 88.61, 82.59, 48.08, 45.29, 25.31, 24.65. HRMS: calcd for C_13_H_14_ON [M + H]^+^: 200.1070, found: 200.1068.

*1-Morpholino-3-phenylprop-2-yn-1-one* (**3ad**): Yellow liquid and the yield is 91%; ^1^H NMR (400 MHz, CDCl_3_) δ 7.52 (d, *J* = 7.6 Hz, 2H), 7.43–7.33 (m, 3H), 3.82 (t, *J* = 4.6 Hz, 2H), 3.73 (t, *J* = 4.5 Hz, 2H), 3.68 (s, 4H). ^13^C NMR (101 MHz, CDCl_3_) δ 153.13, 132.30, 130.12, 128.49, 120.19, 91.11, 80.68, 66.82, 66.41, 47.24, 41.90. HRMS: calcd for C13H_14_O_2_N [M + H]^+^: 216.1016, found: 216.1018.

## 4. Conclusions

In summary, we developed an efficient metal-free strategy for the synthesis of aryl alkynamides via C-N bond cleavage using inert tertiary amines and aryl alkynyl acids. This method employs inexpensive and readily available tertiary amines as starting materials and demonstrates broad substrate scope and excellent functional group compatibility. Notably, the aryl alkynamides are obtained in a one-pot manner under mild conditions, offering significant environmental and economic advantages. The successful scale-up of this synthesis demonstrates the potential practical relevance of this reaction.

## Figures and Tables

**Table 1:** Optimization of reaction conditions ^a^.

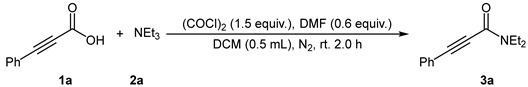		
Entry	Variation from Standard Conditions	Yield ^b^ (%)
1	none	94
2	MeCN as a solvent	84
3	Toluene as a solvent	52
4	Dioxane as a solvent	27
5	DMSO as a solvent	NR
6	MeOH as a solvent	NR
7	without DMF	66
8	DMF (0.2 equiv.)	78
9	DMF (0.4 equiv.)	87
10	DMF (0.8 equiv.)	90
11	0.5 h	47
12	1.0 h	75
13	1.5 h	86
14	2.5 h	93

^a^ Reaction conditions: **1a** (0.2 mmol), **2a** (3.5 equiv.), (COCl)_2_ (1.5 equiv.), DMF (0.6 equiv.), DCM (0.5 mL), rt, under nitrogen atmosphere for 2 h; ^b^ Isolated yields.

## Data Availability

Data are contained within the article and Supplementary Materials.

## Supplementary Materials

The following supporting information can be downloaded at https://www.mdpi.com/article/10.3390/molecules30142955/s1. Experimental details: p. S2, General information and Synthesis of 3-arylpropiolic acids; pp. S3–S6, Experimental characterization data for products; pp. S7–S35, Copies of product 1H NMR, 13C NMR.
