# Supplementary material for: Rapid and One-Pot Synthesis of Aryl Ynamides from Aryl Alkynyl Acids by Metal-Free C-N Cleavage of Tertiary Amines
Source: Molecules. 2025 Jul 13;30(14):2955. doi: 10.3390/molecules30142955 (PMC12300234; doi:10.3390/molecules30142955)

Supporting Information

Rapid and one-pot synthesis of aryl ynamides from aryl alkynyl acids by metal-free C-N cleavage of tertiary amines

Yong Liu, Xiaoyong Liu, Hongwei Li and Shengmei Guo

CONTENTS

1. General information
2. Synthesis of 3-arylpropiolic acids
3. Experimental characterization data for products
4. Copies of product ^{1}H NMR, ^{13}C NMR
5. Reference

1. General information

All commercially available reagent grade chemicals were purchased from Adamas, Aldrich, Accela, Alfa Aesar, TCI and used as received without further purification unless otherwise stated. ^{1}H NMR, ^{13}C NMR were recorded in $CDCl_3$ on a Bruker Avance III 400 spectrometer with TMS as internal standard (400 MHz ^{1}H, 101 MHz ^{13}C) at room temperature, the chemical shifts (δ) were expressed in ppm and J values were given in Hz. HRMS was performed by the Analysis and Testing Center, Nanchang University. The following abbreviations are used to indicate the multiplicity: singlet (s), doublet (d), triplet (t), quartet (q), doublet of doublets (dd), doublet of triplets (dt), and multiplet (m). All first-order splitting patterns were assigned based on the appearance of the multiplet. Splitting patterns that could not be easily interpreted were designated as multiplet (m). Column chromatography was performed on silica gel (200-300 mesh).

2. Synthesis of 3-arylpropiolic acids [1]

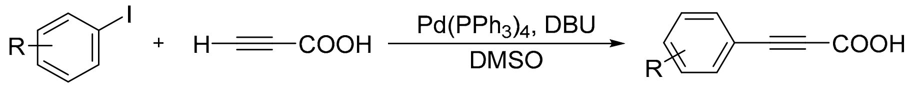

A 25-mL round bottom flask equipped with a magnetic stir bar was sequentially charged with aryliodide (5.0 mmol), DBU (1.80 mL, 12 mmol), $Pd(PPh_3)_4$ (144.4 mg, 2.5 mol %) and DMSO (6 mL). A solution of propiolic acid (420 mg, 6.0 mmol) in DMSO (6 mL) was added to the flask. The mixture was stirred at room temperature for 12 h. After the reaction was complete, EtOAc (20 mL) was poured into the reaction mixture. The reaction mixture was extracted with a saturated aqueous $NaHCO_3$ solution. The aqueous layer was separated, acidified to pH 2.0 by adding cold HCl aqueous solution (1 N), and extracted with CH_2Cl_2. The combined organic layers were dried with anhydrous Na_2SO_4 and filtered, and the solvent was removed under reduced pressure. The resulting crude product was purified by column chromatography on silica gel (eluent: petroleum ether/ethyl acetate = 4:1, with 1% v/v HOAc) to afford 3-arylpropiolic acids.

[1] Kyungho P, Thiruvengadam P, Ayoung P, Sunwoo L. *Tetrahedron Lett*, 2012, 53: 733-737.

3. Experimental characterization data for products

N,N-Diethyl-3-phenylpropiolamide (3a): Yellow liquid and the yield is 94%;

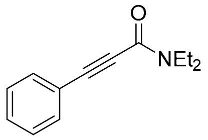

^{1}H NMR (400 MHz, $CDCl_3$) δ 7.50 – 7.45 (m, 2H), 7.38 – 7.27 (m, 3H), 3.61 (q, *J* = 7.1 Hz, 2H), 3.42 (q, *J* = 7.1 Hz, 2H), 1.22 (t, *J* = 7.1 Hz, 3H), 1.12 (t, *J* = 7.2 Hz, 3H). **^{13}C NMR** (101 MHz, $CDCl_3$) δ 153.71, 132.05, 129.66, 128.27, 120.49, 88.71, 81.73, 43.39, 39.10, 14.18, 12.64. **HRMS**: calcd for $C_{13}H_{16}ON$ $[M+H]^+$: 202.1226, found: 202.1222.

N,N-Diethyl-3-(p-tolyl)propiolamide (3b): Yellow liquid and the yield is 84%;

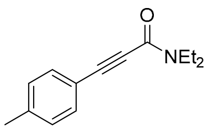

^{1}H NMR (400 MHz, $CDCl_3$) δ 7.42 (d, *J* = 8.3 Hz, 2H), 7.15 (d, *J* = 7.8 Hz, 2H), 3.69 – 3.61 (m, 2H), 3.46 (q, *J* = 7.1 Hz, 2H), 2.36 (s, 3H), 1.27 (t, *J* = 7.1 Hz, 3H), 1.16 (t, *J* = 7.1 Hz, 3H). **^{13}C NMR** (101 MHz, $CDCl_3$) δ 154.09, 140.29, 132.22, 129.20, 117.57, 89.34, 81.44, 43.54, 39.22, 21.58, 14.34, 12.83. **HRMS**: calcd for $C_{14}H_{18}ON$ $[M+H]^+$: 216.1383, found: 216.1329.

3-(4-(Tert-butyl)phenyl)-N,N-diethylpropiolamide (3c): Yellow liquid and the yield is 87%;

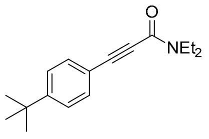

^{1}H NMR (400 MHz, $CDCl_3$) δ 7.46 (d, *J* = 8.3 Hz, 2H), 7.37 (d, *J* = 8.5 Hz, 2H), 3.65 (q, *J* = 7.1, 6.6 Hz, 2H), 3.46 (q, *J* = 7.1 Hz, 2H), 1.30 (s, 9H), 1.26 (t, *J* = 7.0 Hz, 3H), 1.17 (t, *J* = 7.1 Hz, 3H). **^{13}C NMR** (101 MHz, $CDCl_3$) δ 154.05, 153.31, 132.05, 125.43, 117.58, 89.24, 81.41, 43.52, 39.22, 34.84, 30.97, 14.30, 12.79. **HRMS**: calcd for $C_{17}H_{24}ON$ $[M+H]^+$: 258.1852, found: 258.1845.

N,N-Diethyl-3-(4-methoxyphenyl)propiolamide (3d): Yellow liquid and the yield is 95%;

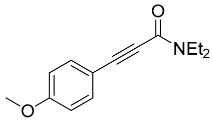

^{1}H NMR (400 MHz, $CDCl_3$) δ 7.47 (d, *J* = 7.0 Hz, 2H), 6.86 (d, *J* = 6.9 Hz, 2H), 3.81 (s, 3H), 3.64 (q, *J* = 7.2 Hz, 2H), 3.45 (q, *J* = 7.1 Hz, 2H), 1.26 (t, *J* = 7.1 Hz, 3H), 1.16 (t, *J* = 8.0 Hz, 3H). **^{13}C NMR** (101 MHz, $CDCl_3$) δ 160.82, 154.23, 134.02, 114.12, 112.59, 89.46, 81.12, 55.30, 43.52, 39.20, 14.33, 12.84. **HRMS**: calcd for $C_{14}H_{18}O_2N$ $[M+H]^+$: 232.1332, found: 232.1328.

3-(4-Bromophenyl)-N,N-diethylpropiolamide (3e): Yellow liquid and the yield is 81%;

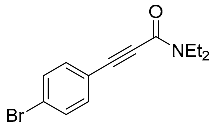

^{1}H NMR (400 MHz, $CDCl_3$) δ 7.49 (d, *J* = 8.3 Hz, 2H), 7.37 (d, *J* = 8.3 Hz, 2H), 3.63 (q, *J* = 7.2 Hz, 2H), 3.46 (q, *J* = 7.2 Hz, 2H), 1.26 (t, *J* = 7.2 Hz, 3H), 1.16 (t, *J* = 7.2 Hz, 3H). **^{13}C NMR** (101 MHz, $CDCl_3$) δ 153.64, 133.60, 131.79, 124.41, 119.59, 87.76, 82.79, 43.54, 39.28, 14.36, 12.77. **HRMS**: calcd for $C_{13}H_{15}BrON$ $[M+H]^+$: 280.0332, found: 280.0329.

3-(4-Cyanophenyl)-N,N-diethylpropiolamide (3f): Yellow liquid and the yield is 38%;

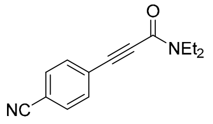

^{1}H NMR (400 MHz, $CDCl_3$) δ 7.63 (q, *J* = 8.4, 7.9 Hz, 4H), 3.64 (q, *J* = 7.1 Hz, 2H), 3.47 (q, *J* = 7.0 Hz, 2H), 1.27 (t, *J* = 7.2 Hz, 3H), 1.18 (t, *J* = 7.2 Hz, 3H). **^{13}C NMR** (101 MHz, $CDCl_3$) δ 153.11, 132.66, 132.12, 125.53, 117.95, 113.21, 86.53, 85.22, 43.58, 39.40, 14.39, 12.73. **HRMS**: calcd for $C_{14}H_{15}ON_2$ $[M+H]^+$: 227.1179, found: 227.1174.

N,N-Diethyl-3-(4-formylphenyl)propiolamide (3g): Yellow liquid and the yield is 32%;

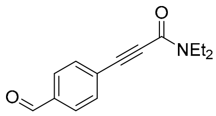

^{1}H NMR (400 MHz, $CDCl_3$) δ 10.03 (s, 1H), 7.88 (d, *J* = 8.5 Hz, 2H), 7.68 (d, *J* = 8.2 Hz, 2H), 3.66 (q, *J* = 7.1 Hz, 2H), 3.48 (q, *J* = 7.2 Hz, 2H), 1.29 (t, *J* = 7.2 Hz, 3H), 1.18 (t, *J* = 7.2 Hz, 3H). **^{13}C NMR** (101 MHz, $CDCl_3$) δ 191.20, 153.34,

136.49, 132.76, 129.52, 126.73, 87.43, 84.83, 43.59, 39.37, 14.42, 12.78. **HRMS**: calcd for $C_{14}H_{16}O_2N$ $[M+H]^+$: 230.2870, found: 230.2867.

3-(4-Acetylphenyl)-N,N-diethylpropiolamide (3h): Yellow liquid and the yield is 55%;

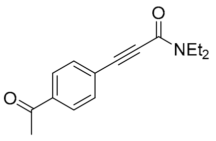

^{1}H NMR (400 MHz, $CDCl_3$) δ 7.93 (d, *J* = 8.4 Hz, 2H), 7.60 (d, *J* = 8.5 Hz, 2H), 3.65 (q, *J* = 7.0 Hz, 2H), 3.47 (q, *J* = 7.1 Hz, 2H), 2.60 (s, 3H), 1.27 (t, *J* = 7.2 Hz, 3H), 1.17 (t, *J* = 7.2 Hz, 3H). **^{13}C NMR** (101 MHz, $CDCl_3$) δ 197.05, 153.44, 137.40, 132.35, 128.21, 125.34, 87.65, 84.31, 43.58, 39.33, 26.60, 14.36, 12.74. **HRMS**: calcd for $C_{15}H_{18}O_2N$ $[M+H]^+$: 244.1332, found: 244.1328.

N,N-Diethyl-3-(o-tolyl)propiolamide (3i): Yellow liquid and the yield is 86%

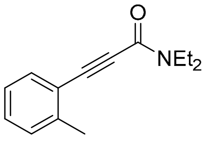

^{1}H NMR (400 MHz, $CDCl_3$) δ 7.50 (d, *J* = 7.8 Hz, 1H), 7.28 (t, *J* = 7.5 Hz, 1H), 7.21 (d, *J* = 7.6 Hz, 1H), 7.16 (t, *J* = 7.5 Hz, 1H), 3.66 (q, *J* = 6.7 Hz, 2H), 3.47 (q, *J* = 7.1, 6.6 Hz, 2H), 2.46 (s, 3H), 1.26 (t, *J* = 7.2 Hz, 3H), 1.17 (t, *J* = 7.2 Hz, 3H). **^{13}C NMR** (101 MHz, $CDCl_3$) δ 154.03, 141.06, 132.88, 129.81, 129.57, 125.68, 120.52, 87.98, 85.67, 43.52, 39.28, 20.58, 14.40, 12.81. **HRMS**: calcd for $C_{14}H_{18}ON$ $[M+H]^+$: 216.1383, found: 216.1328.

N,N-Diethyl-3-(2-methoxyphenyl)propiolamide (3j): Yellow liquid and the yield is 90%

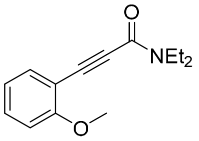

^{1}H NMR (400 MHz, $CDCl_3$) δ 7.48 (d, *J* = 7.6 Hz, 1H), 7.35 (t, *J* = 7.6 Hz, 1H), 6.91 (t, *J* = 7.6 Hz, 1H), 6.87 (d, *J* = 8.4 Hz, 1H), 3.85 (s, 3H), 3.70 (q, *J* = 7.1 Hz, 2H), 3.46 (q, *J* = 7.1 Hz, 2H), 1.26 (t, *J* = 7.1 Hz, 3H), 1.15 (t, *J* = 7.1 Hz, 3H). **^{13}C NMR** (101 MHz, $CDCl_3$) δ 161.08, 154.17, 134.13, 131.41, 120.41, 110.57, 109.96, 86.00, 85.53, 55.62, 43.50, 39.17, 14.28, 12.83. **HRMS**: calcd for $C_{14}H_{18}O_2N$ $[M+H]^+$: 232.1332, found: 232,1328.

3-(2-Chlorophenyl)-N,N-diethylpropiolamide (3k): Yellow liquid and the yield is 87%

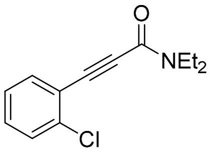

^{1}H NMR (400 MHz, $CDCl_3$) δ 7.59 (d, *J* = 7.4 Hz, 1H), 7.40 (t, *J* = 5.6 Hz, 1H), 7.32 (t, *J* = 7.4 Hz, 1H), 7.25 (d, *J* = 7.4 Hz, 1H), 3.71 (q, *J* = 7.0 Hz, 2H), 3.46 (q, *J* = 7.1 Hz, 2H), 1.26 (t, *J* = 7.1 Hz, 3H), 1.26 (t, *J* = 7.1 Hz, 3H). **^{13}C NMR** (101 MHz, $CDCl_3$) δ 153.61, 136.67, 134.42, 130.89, 129.35, 126.65, 120.88, 86.44, 85.13, 43.56, 39.34, 14.45, 12.78. **HRMS**: calcd for $C_{13}H_{15}ClON$ $[M+H]^+$: 236.0837, found: 286.0834.

N,N-Diethyl-3-(naphthalen-2-yl)propiolamide (3l): Yellow liquid and the yield is 70%

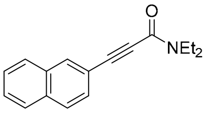

^{1}H NMR (400 MHz, $CDCl_3$) δ 8.08 (s, 1H), 7.81 (dd, *J* = 7.3, 4.4 Hz, 3H), 7.56 – 7.48 (m, 3H), 3.70 (q, *J* = 7.1 Hz, 2H), 3.49 (q, *J* = 7.2 Hz, 2H), 1.31 (t, *J* = 7.1 Hz, 3H), 1.19 (t, *J* = 7.2 Hz, 3H). **^{13}C NMR** (101 MHz, $CDCl_3$) δ 153.94, 133.39, 132.99, 132.60, 128.20, 128.05, 127.92, 127.76, 127.46, 126.81, 117.87, 89.37, 82.06, 43.59, 39.27, 14.40, 12.82. **HRMS**: calcd for $C_{17}H_{18}ON$ $[M+H]^+$: 252.3370, found: 252.3368.

N,N-Diethyl-3-(thiophen-2-yl)propiolamide (3m): Yellow liquid and the yield is 83%

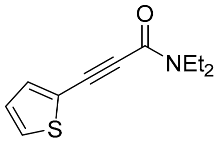

^{1}H NMR (400 MHz, $CDCl_3$) δ 7.38 (t, *J* = 3.4 Hz, 2H), 7.02 (t, *J* = 4.3 Hz, 1H), 3.61 (q, *J* = 7.2 Hz, 2H), 3.45 (q, *J* = 7.2 Hz, 2H), 1.25 (t, *J* = 7.1 Hz, 3H), 1.15 (t, *J* = 7.2 Hz, 3H). **^{13}C NMR** (101 MHz, $CDCl_3$) δ 153.70, 134.79, 129.72, 127.30, 120.36, 85.83, 82.69, 43.47, 39.21, 14.34, 12.79. **HRMS**: calcd for $C_{11}H_{14}OSN$ $[M+H]^+$: 208.2990, found: 208.2986.

3-Phenyl-N,N-dipropylpropiolamide (3q): Yellow liquid and the yield is 73%

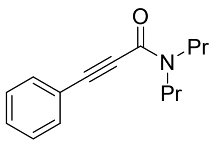

^{1}H NMR (400 MHz, $CDCl_3$) δ 7.52 (d, *J* = 7.3 Hz, 2H), 7.42 – 7.33 (m, 3H), 3.56 (t, *J* = 7.4 Hz, 2H), 3.36 (t, *J* = 7.4 Hz, 2H), 1.73 - 1.66 (m, 2H),1.63 - 1.56 (m, 2H), 0.96 (t, *J* = 7.4 Hz, 3H), 0.92 (t, *J* = 7.4 Hz, 3H). **^{13}C NMR** (101 MHz, $CDCl_3$) δ 154.53, 132.25, 129.82, 128.46, 120.78, 89.26, 82.11, 50.84, 46.50, 22.17, 20.69, 11.32, 11.23. **HRMS**: calcd for $C_{15}H_{20}ON$ [M+H] $^+$: 230.1539, found: 230.1536.

N,N-Dihexyl-3-phenylpropiolamide (3r): Yellow liquid, and the yield is 94%

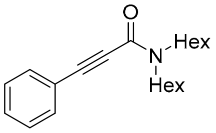

^{1}H NMR (400 MHz, $CDCl_3$) δ 7.52 (d, *J* = 7.0 Hz, 2H), 7.42 - 7.33 (m, 3H), 3.58 (t, *J* = 7.5 Hz, 2H), 3.38 (t, *J* = 7.5 Hz, 2H), 1.69 – 1.61 (m, 2H), 1.60 – 1.53 (m, 2H), 1.37 – 1.25 (m, 12H), 0.90 – 0.84 (m, 6H). **^{13}C NMR** (101 MHz, $CDCl_3$) δ 154.33, 132.21, 129.76, 128.42, 120.79, 89.13, 82.14, 54.09, 49.11, 44.81, 31.78, 31.53, 28.82, 27.41, 26.59, 26.38, 22.51, 13.96, 13.90. **HRMS**: calcd for $C_{21}H_{32}ON$ [M+H] $^+$: 314.2478, found: 314.2474.

N,N-Dioctyl-3-phenylpropiolamide (3s): Yellow liquid and the yield is 80%

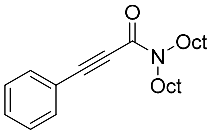

^{1}H NMR (400 MHz, $CDCl_3$) δ 7.52 (d, *J* = 7.3 Hz, 2H), 7.41 – 7.41 (m, 3H), 3.58 (t, *J* = 7.5 Hz, 2H), 3.38 (t, *J* = 7.5 Hz, 2H), 1.65 (t, *J* = 7.3 Hz, 2H), 1.56 (t, *J* = 7.4 Hz, 2H), 1.34 – 1.23 (m, 20H), 0.89 – 0.83 (m, 6H). **^{13}C NMR** (101 MHz, $CDCl_3$) δ 154.40, 132.26, 129.80, 128.46, 120.83, 89.22, 82.17, 53.91, 49.18, 44.87, 31.77, 31.74, 29.34, 29.26, 29.20, 28.89, 27.48, 26.98, 26.75, 22.61, 22.58, 14.05, 14.03. **HRMS**: calcd for $C_{25}H_{40}ON$ [M+H] $^+$: 370.3104, found: 370.3100.

N,N-Dibenzyl-3-phenylpropiolamide (3t): Yellow liquid and the yield is 33%

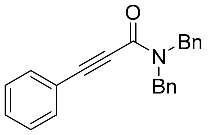

^{1}H NMR (400 MHz, $CDCl_3$) δ 7.52 (s, 1H), 7.50 (s, 1H), 7.43 – 7.24 (m, 13H), 4.76 (s, 2H), 4.57 (s, 2H). **^{13}C NMR** (101 MHz, $CDCl_3$) δ 154.98, 136.19, 136.03, 132.41, 130.09, 128.85, 128.66, 128.47, 128.43, 127.93, 127.69, 127.62, 120.31, 90.80, 81.60, 51.41, 46.34. **HRMS**: calcd for $C_{23}H_{20}ON$ [M+H] $^+$: 326.1539, found: 326.1534.

N,N-Diallyl-3-phenylpropiolamide (3u): Yellow liquid and the yield is 68%

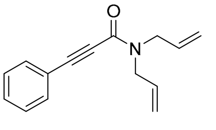

^{1}H NMR (400 MHz, $CDCl_3$) δ 7.53 (d, *J* = 7.2 Hz, 2H), 7.44 – 7.32 (m, 3H), 5.89 – 5.71 (m, 2H), 5.27 – 5.15 (m, 4H), 4.22 (d, *J* = 5.8 Hz, 2H), 4.06 (d, *J* = 6.0 Hz, 2H). **^{13}C NMR** (101 MHz, $CDCl_3$) δ 154.41, 132.69, 132.33, 132.09, 129.99, 128.45, 120.43, 118.07, 117.94, 89.87, 81.47, 50.72, 46.37. **HRMS**: calcd for $C_{15}H_{16}ON$ [M+H] $^+$: 226.1226, found: 226.1221.

N,N-Dimethyl-3-phenylpropiolamide (3v): Yellow liquid and the yield is 87%

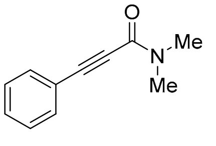

^{1}H NMR (400 MHz, $CDCl_3$) δ 7.53 (d, *J* = 8.0 Hz, 2H), 7.43 – 7.32 (m, 3H), 3.28 (s, 3H), 3.02 (s, 3H). **^{13}C NMR** (101 MHz, $CDCl_3$) δ 154.59, 132.28, 129.91, 128.45, 120.56, 90.12, 81.53, 38.34, 34.13. **HRMS**: calcd for $C_{11}H_{12}ON$ [M+H] $^+$: 174.0913, found: 174.0908.

N-Ethyl-N-isopropyl-3-phenylpropiolamide (3w): Yellow liquid and the yield is 95%

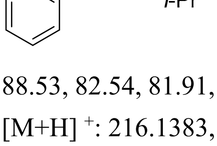

^{1}H NMR (400 MHz, $CDCl_3$) δ 7.52 (d, *J* = 6.8 Hz, 2H), 7.53 – 7.32 (m, 3H), 4.74 – 4.65 (m, 1H), 3.55 (q, *J* = 7.2 Hz, 1H), 3.34 (q, *J* = 7.0 Hz, 1H), 1.32 (t, *J* = 7.4 Hz, 1H), 1.25 (d, *J* = 7.3 Hz, 4H), 1.19 (d, *J* = 7.2 Hz, 4H). **^{13}C NMR** (101 MHz, $CDCl_3$) δ 154.35, 153.85, 132.20, 132.18, 129.75, 129.70, 128.40, 128.37, 89.35, 88.53, 82.54, 81.91, 50.52, 45.33, 39.22, 35.36, 21.21, 20.30, 16.58, 14.45. **HRMS**: calcd for $C_{14}H_{18}ON$ [M+H] $^+$: 216.1383, found: 216.1379.

N-Isopropyl-N-methyl-3-phenylpropiolamide (3x): Yellow liquid and the yield is 23%

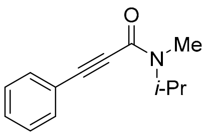

^{1}H NMR (400 MHz, $CDCl_3$) δ 7.53 (d, *J* = 6.7 Hz, 2H), 7.42 – 7.33 (m,3H), 4.89 – 4.71 (m, 1H), 3.12 (s, 1H), 2.86 (s, 2H), 1.24 (d, *J* = 6.7 Hz, 4H), 1.15 (d, *J* = 6.8 Hz, 2H). **^{13}C NMR** (101 MHz, $CDCl_3$) δ 154.31, 154.09, 132.28, 129.83, 128.44, 120.72, 90.03, 89.95, 82.16, 81.50, 50.09, 43.96, 29.79, 25.57, 20.36, 19.22. **HRMS**: calcd for $C_{13}H_{16}ON$ $[M+H]^+$: 202.1226, found: 202.1222.

3-Phenyl-1-(piperidin-1-yl)prop-2-yn-1-one (3aa): Yellow liquid and the yield is 68%

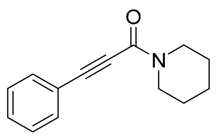

^{1}H NMR (400 MHz, $CDCl_3$) δ 7.54 (d, *J* = 7.4 Hz, 2H), 7.42 – 7.33 (m, 3H), 3.77 (t, *J* = 5.1 Hz, 2H), 3.62 (t, *J* = 5.5 Hz, 2H), 1.66 (t, *J* = 7.4 Hz, 4H), 1.61 – 1.55 (m, 2H). **^{13}C NMR** (101 MHz, $CDCl_3$) δ 152.89, 132.26, 129.81, 128.42, 120.68, 90.20, 81.41, 48.17, 42.32, 26.40, 25.34, 24.48. **HRMS**: calcd for $C_{14}H_{16}ON$ $[M+H]^+$: 214.1226, found: 214.1223.

1-(4-Chloropiperidin-1-yl)-3-phenylprop-2-yn-1-one (3ab): Yellow liquid and the yield is 67%

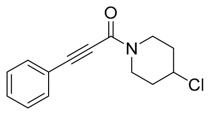

^{1}H NMR (400 MHz, $CDCl_3$) δ 7.56 – 7.51 (m, 2H), 7.45 – 7.33 (m, 3H), 4.36 – 4.31 (m, 1H), 4.07 – 3.98 (m, 1H), 3.88 – 3.79 (m, 2H), 3.77 – 3.69 (m, 1H), 2.18 – 2.02 (m, 2H), 1.99 – 1.83 (m, 2H). **^{13}C NMR** (101 MHz, $CDCl_3$) δ 152.94, 132.32, 130.07, 128.49, 120.32, 90.81, 80.89, 56.17, 44.01, 38.23, 35.12, 34.14. **HRMS**: calcd for $C_{14}H_{15}ClON$ $[M+H]^+$: 248.0837, found: 248.0836.

3-Phenyl-1-(pyrrolidin-1-yl)prop-2-yn-1-one (3ac): Yellow liquid and the yield is 93%

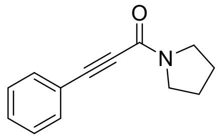

^{1}H NMR (400 MHz, $CDCl_3$) δ 7.52 (d, *J* = 8.3 Hz, 2H), 7.41 – 7.32 (m, 3H), 3.71 (t, *J* = 6.5 Hz, 2H), 3.51 (t, *J* = 6.4 Hz, 2H), 1.99 – 1.90 (m, 4H). **^{13}C NMR** (101 MHz, $CDCl_3$) δ 152.65, 132.31, 129.85, 128.41, 120.56, 88.61, 82.59, 48.08, 45.29, 25.31, 24.65. **HRMS**: calcd for $C_{13}H_{14}ON$ $[M+H]^+$: 200.1070, found: 200.1068.

1-Morpholino-3-phenylprop-2-yn-1-one (3ad): Yellow liquid and the yield is 91%

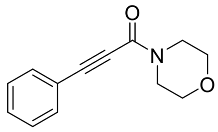

^{1}H NMR (400 MHz, $CDCl_3$) δ 7.52 (d, *J* = 7.6 Hz, 2H), 7.43 – 7.33 (m, 3H), 3.82 (t, *J* = 4.6 Hz, 2H), 3.73 (t, *J* = 4.5 Hz, 2H), 3.68 (s, 4H). **^{13}C NMR** (101 MHz, $CDCl_3$) δ 153.13, 132.30, 130.12, 128.49, 120.19, 91.11, 80.68, 66.82, 66.41, 47.24, 41.90. **HRMS**: calcd for $C_{13}H_{14}O_2N$ $[M+H]^+$: 216.1016, found: 216.1018.

4. Copies of product ^{1}H NMR, ^{13}C NMR

^{1}H NMR of **3a** in $CDCl_3$ (400 MHz, $CDCl_3$)

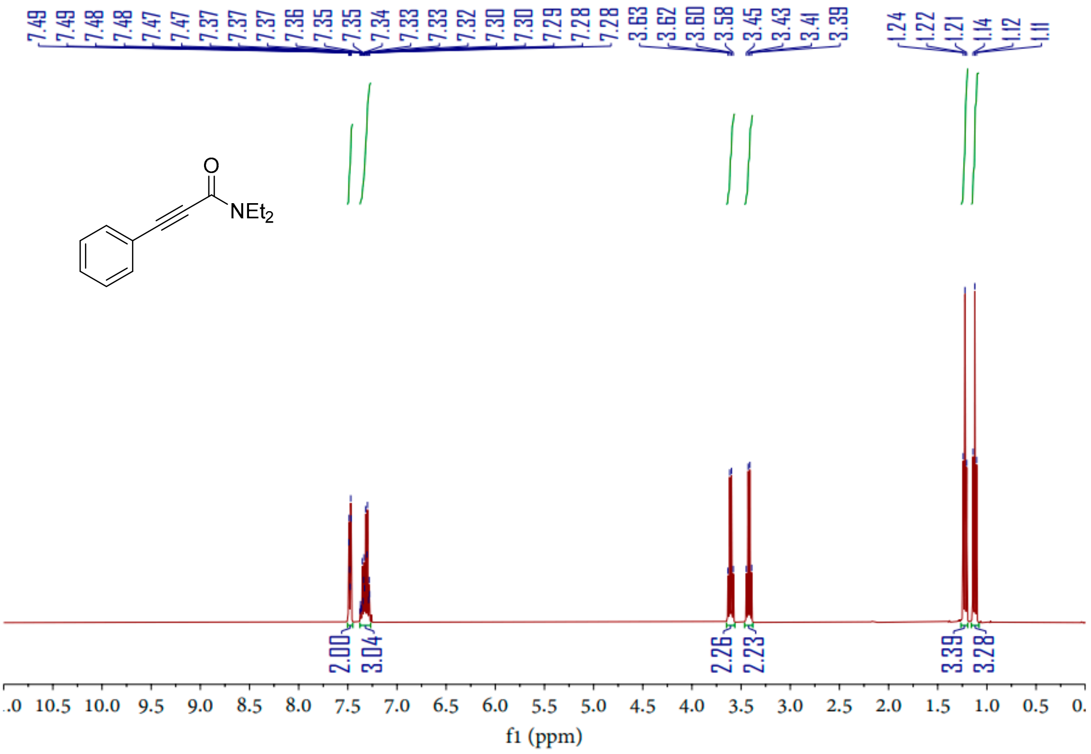

^{13}C NMR of **3a** in $CDCl_3$ (101 MHz, $CDCl_3$)

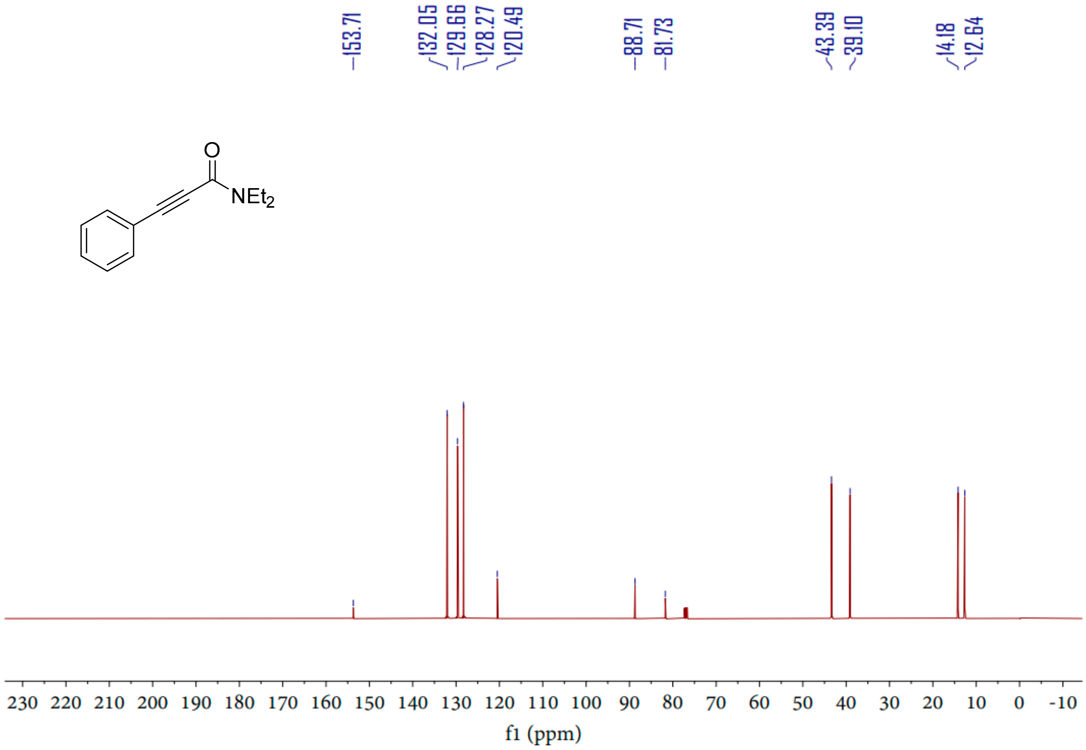

^{1}H NMR of **3b** in $CDCl_3$ (400 MHz, $CDCl_3$)

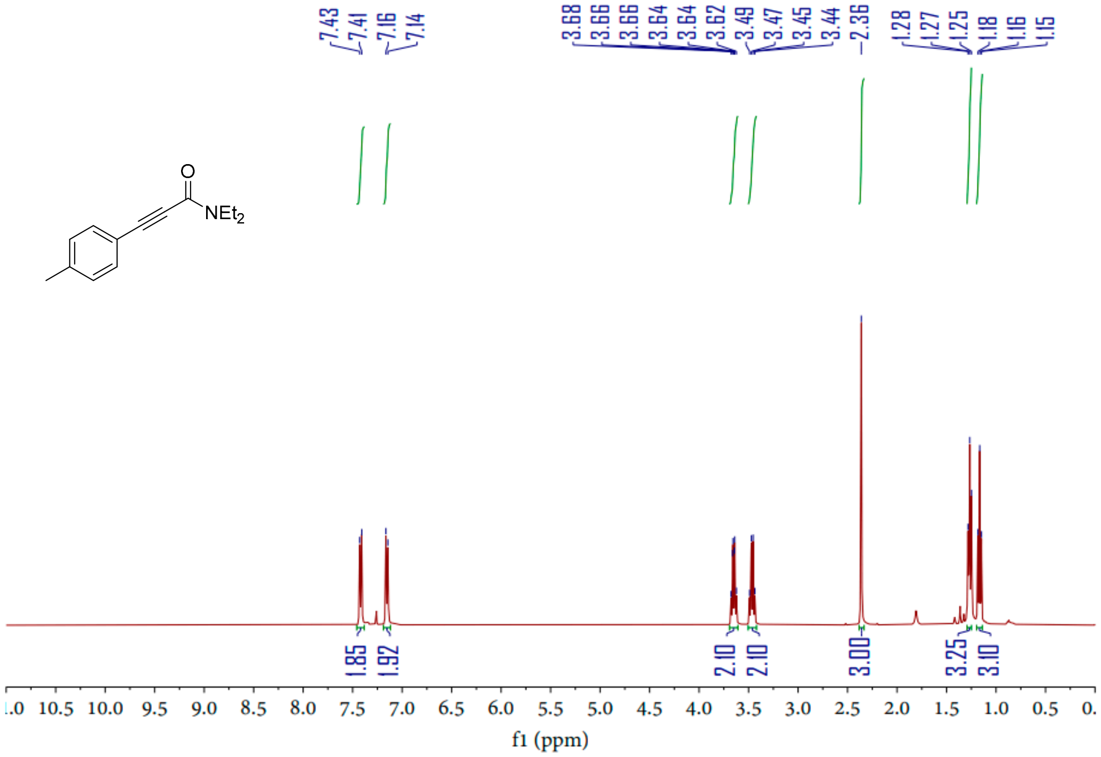

^{13}C NMR of **3b** in $CDCl_3$ (101 MHz, $CDCl_3$)

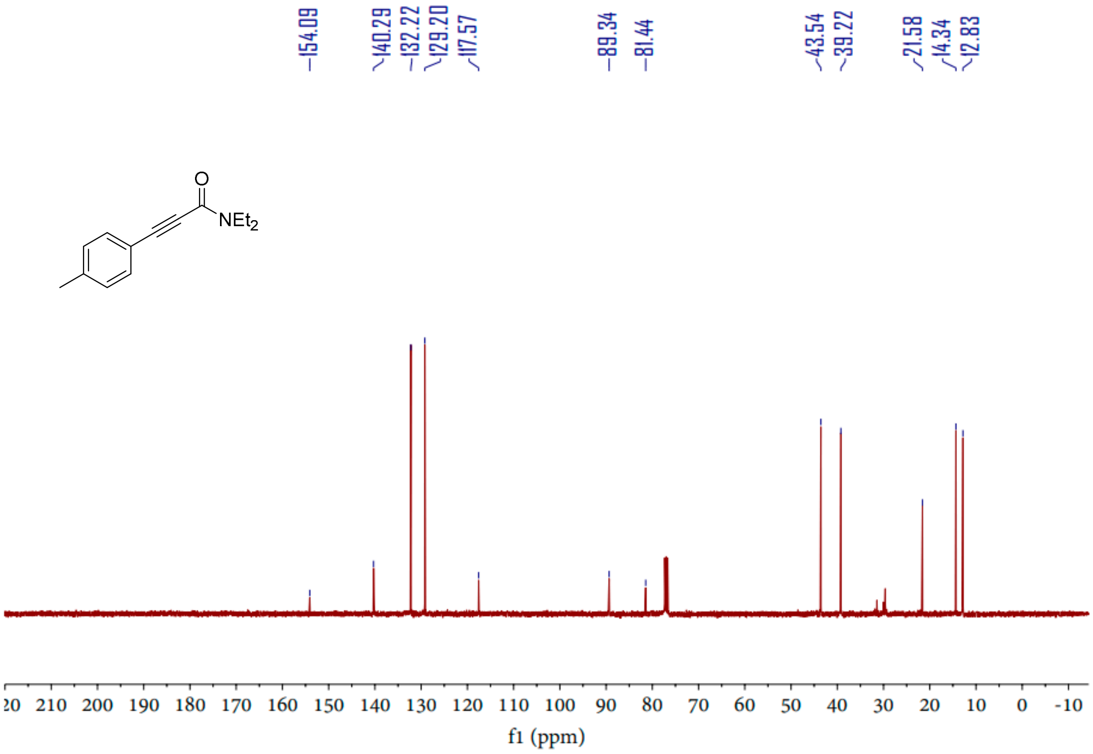

^{1}H NMR of **3c** in $CDCl_3$ (400 MHz, $CDCl_3$)

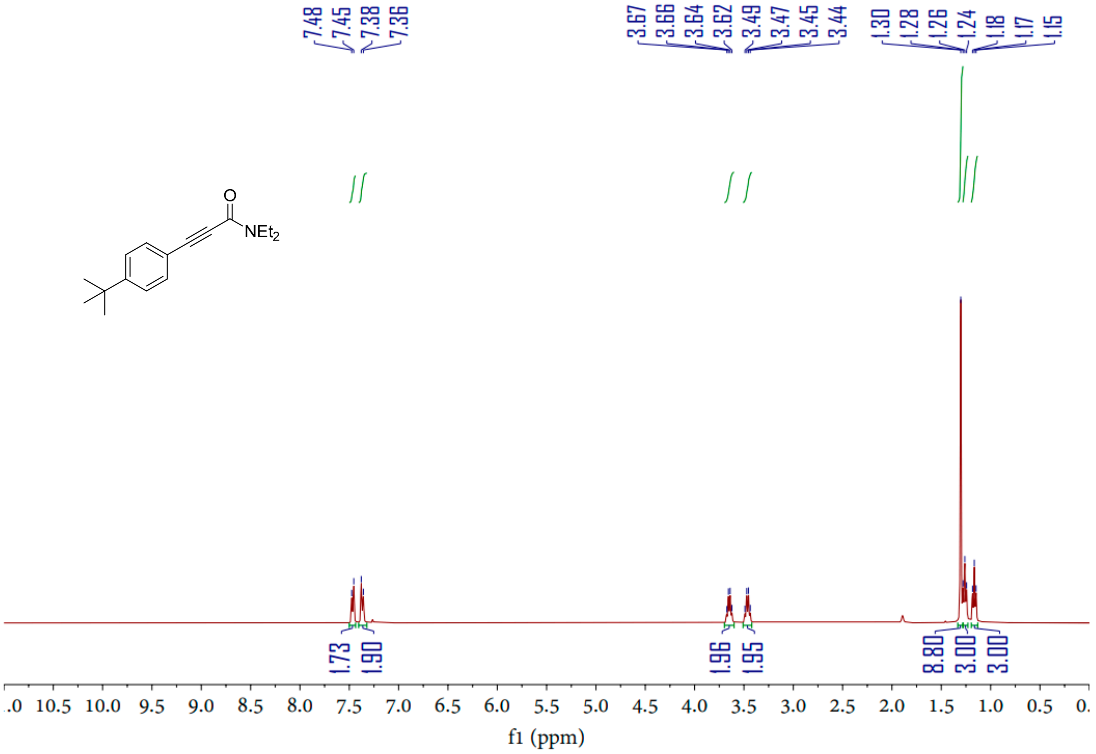

^{13}C NMR of **3c** in $CDCl_3$ (101 MHz, $CDCl_3$)

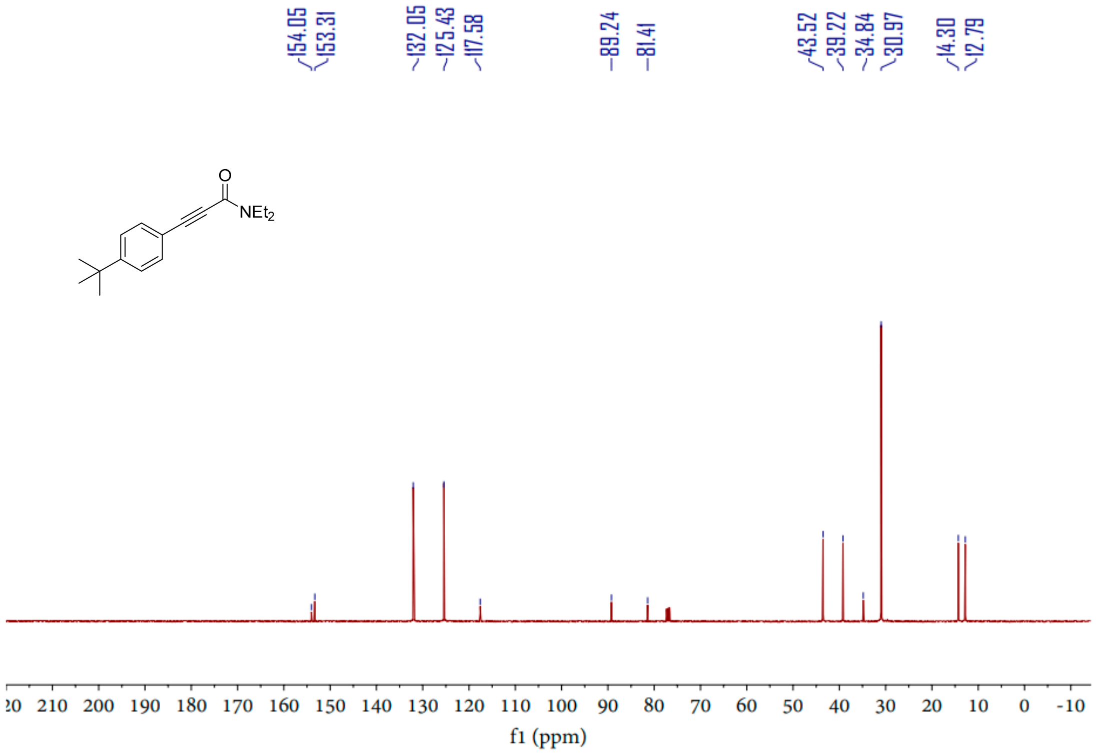

^{1}H NMR of **3d** in $CDCl_3$ (400 MHz, $CDCl_3$)

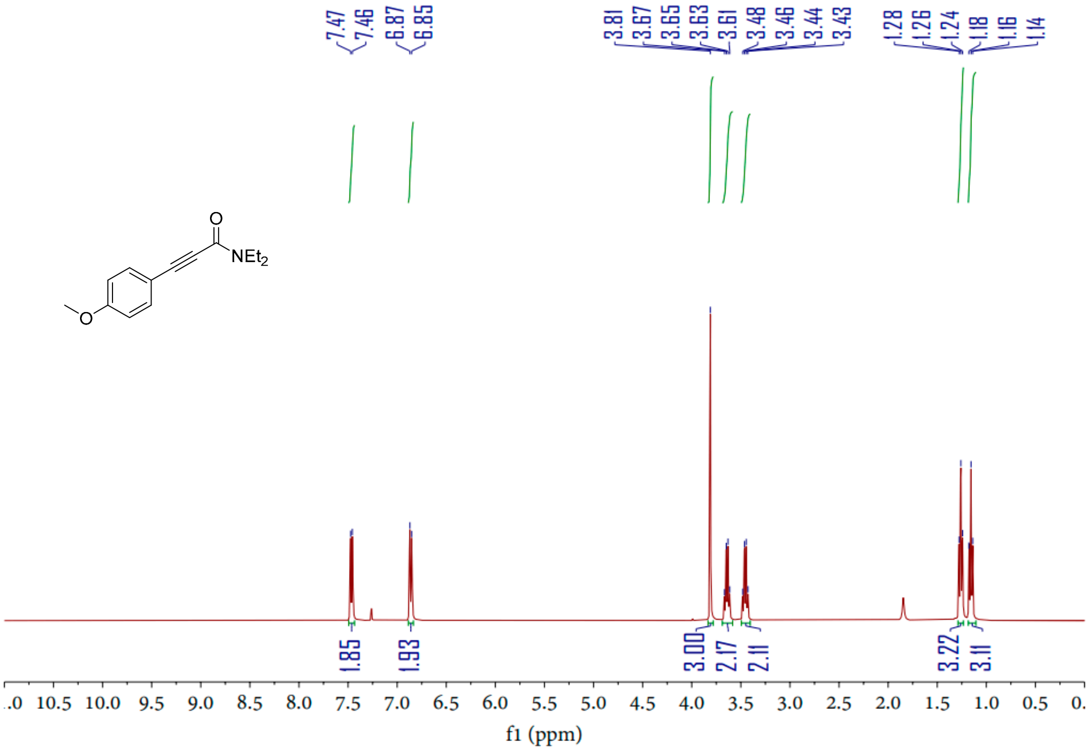

^{13}C NMR of **3d** in $CDCl_3$ (101 MHz, $CDCl_3$)

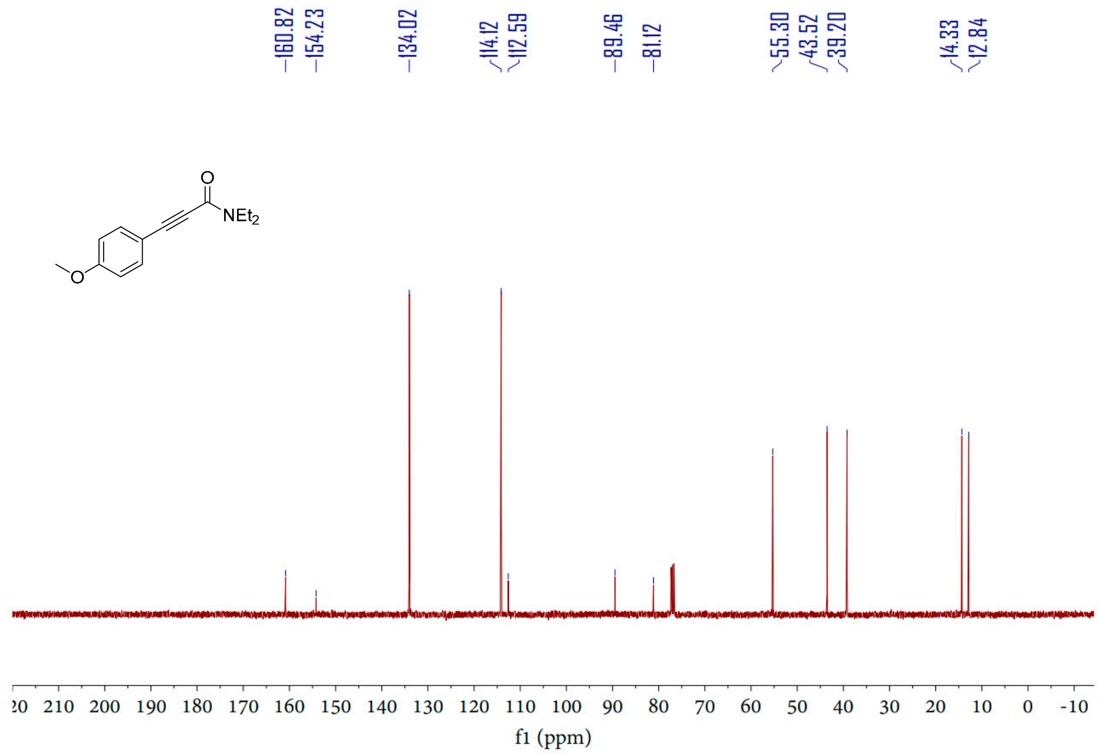

^{1}H NMR of **3e** in $CDCl_3$ (400 MHz, $CDCl_3$)

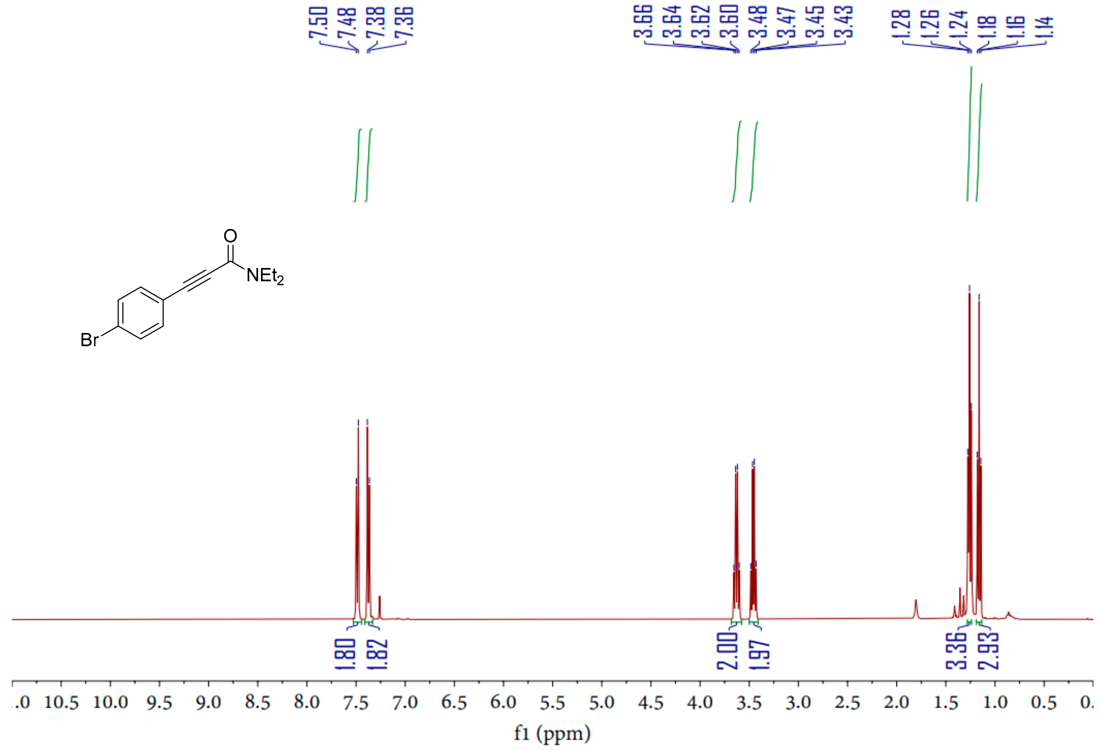

^{13}C NMR of **3e** in $CDCl_3$ (101 MHz, $CDCl_3$)

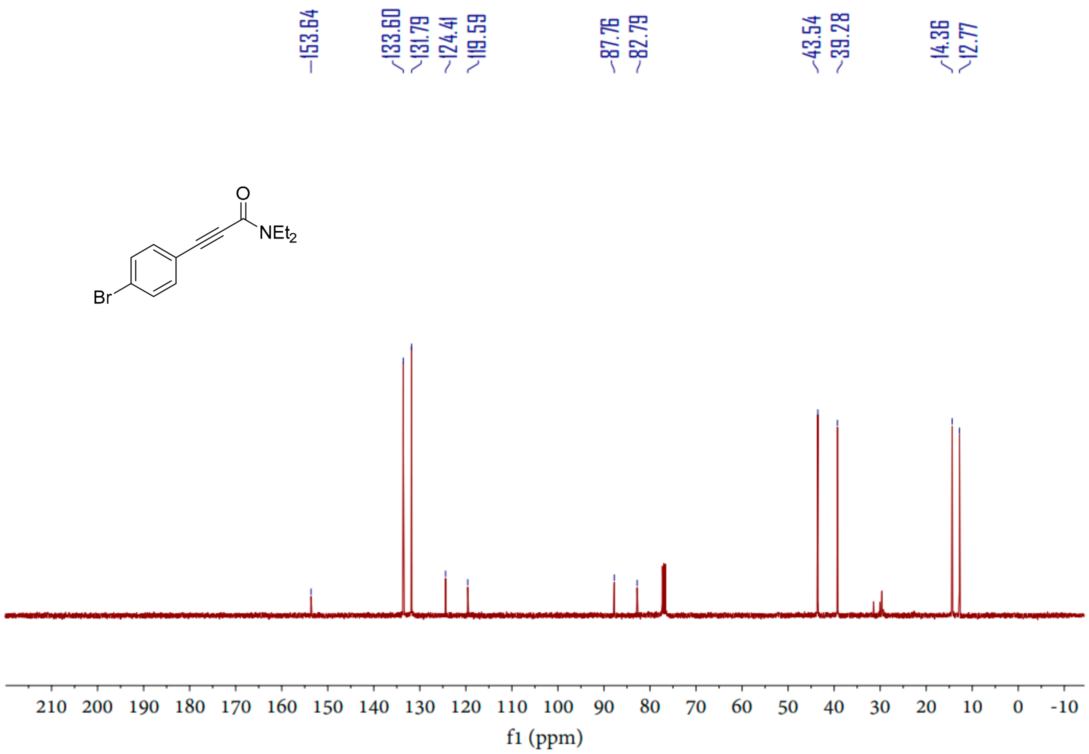

^{1}H NMR of **3f** in $CDCl_3$ (400 MHz, $CDCl_3$)

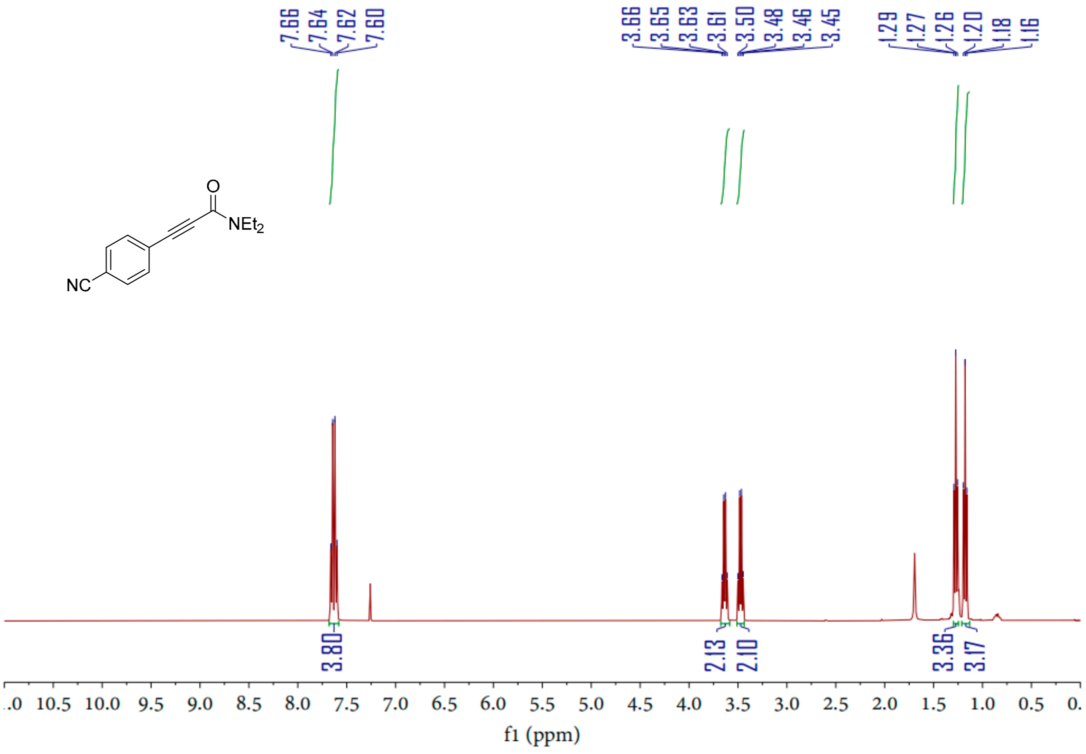

^{13}C NMR of **3f** in $CDCl_3$ (101 MHz, $CDCl_3$)

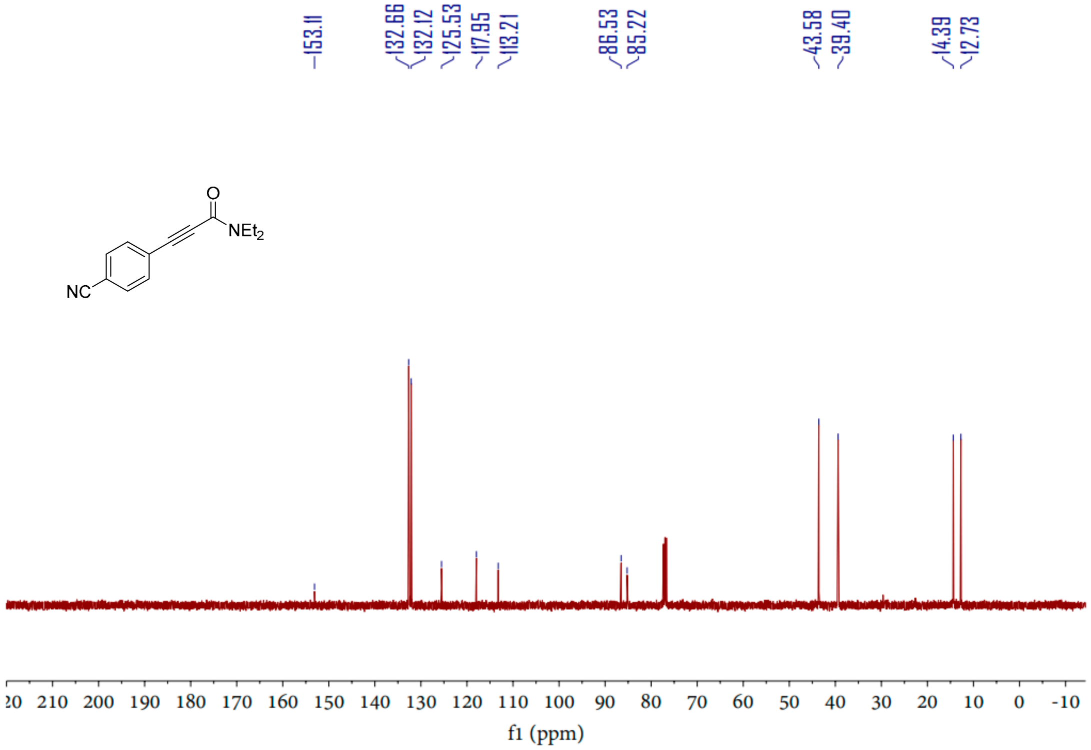

^{1}H NMR of **3g** in $CDCl_3$ (400 MHz, $CDCl_3$)

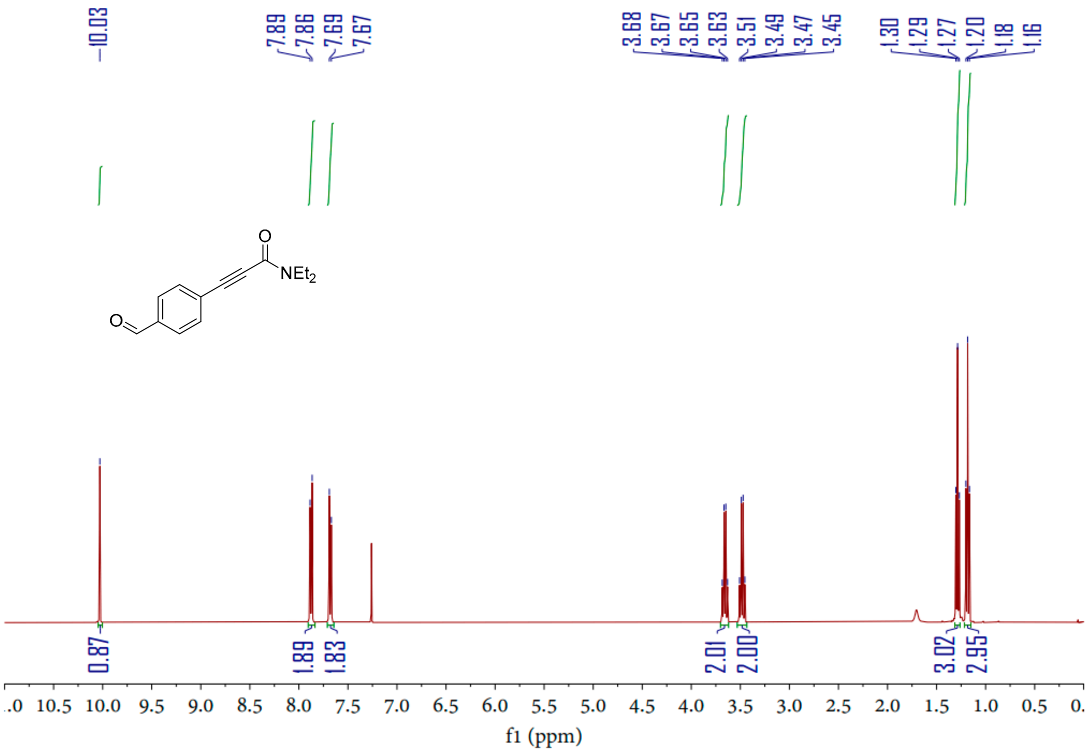

^{13}C NMR of **3g** in $CDCl_3$ (101 MHz, $CDCl_3$)

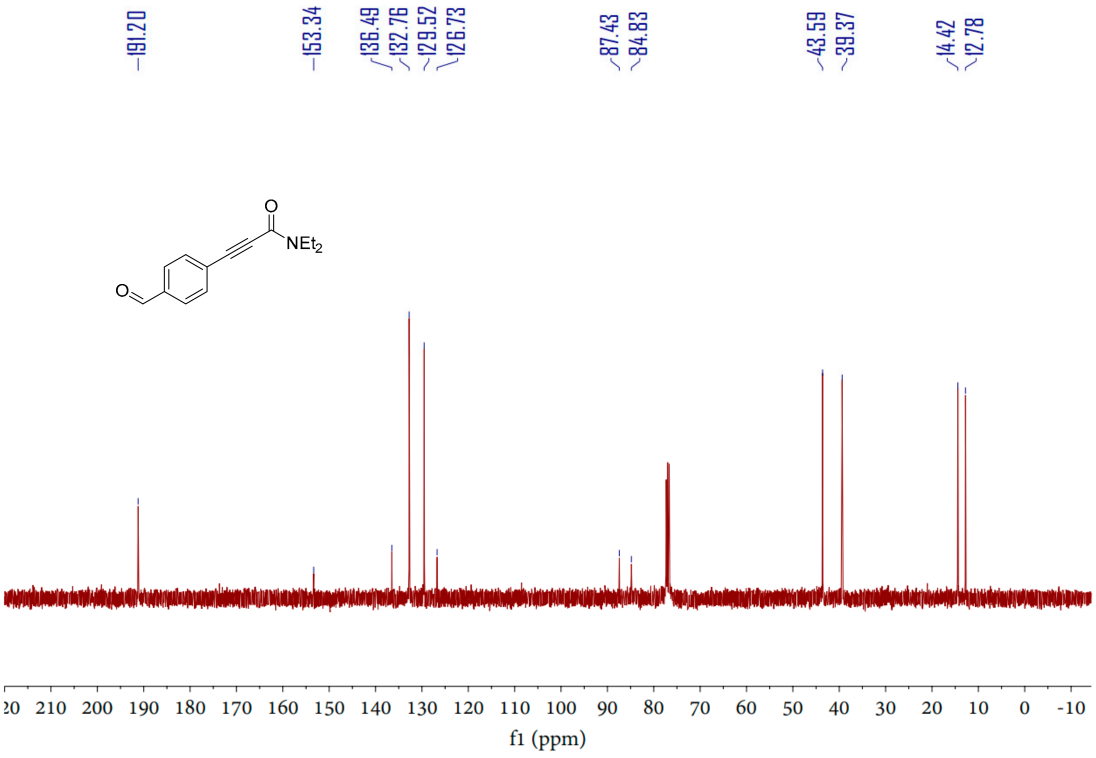

^{1}H NMR of **3h** in $CDCl_3$ (400 MHz, $CDCl_3$)

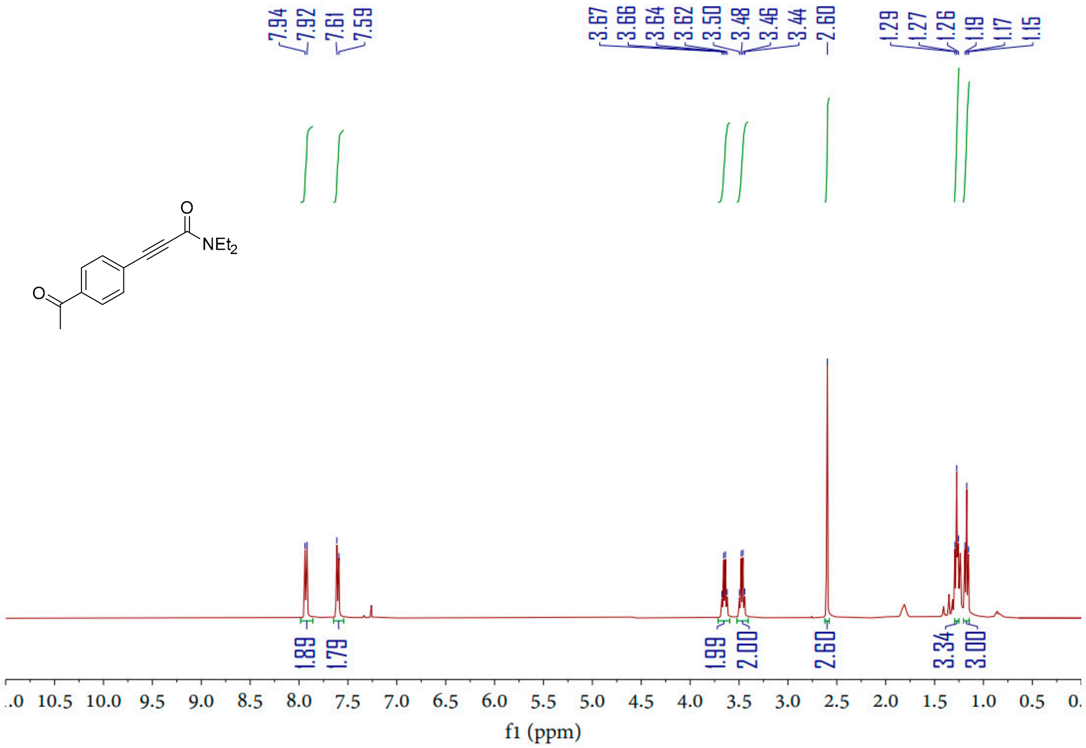

^{13}C NMR of **3h** in $CDCl_3$ (101 MHz, $CDCl_3$)

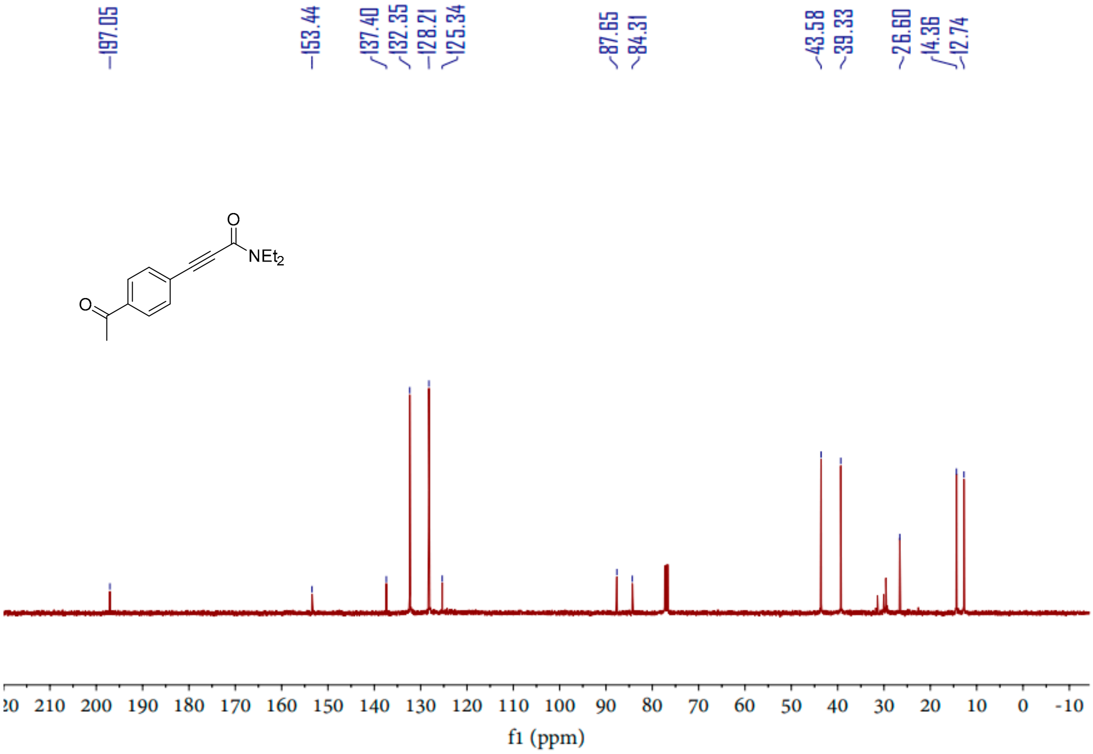

^{1}H NMR of **3i** in $CDCl_3$ (400 MHz, $CDCl_3$)

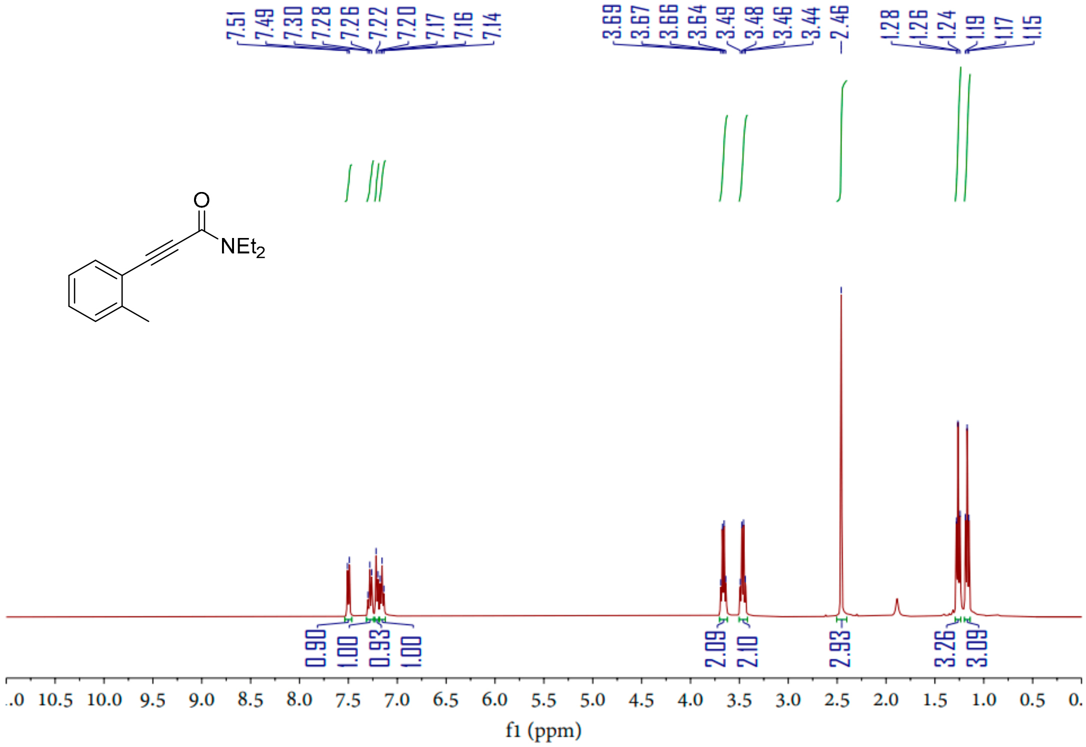

^{13}C NMR of **3i** in $CDCl_3$ (101 MHz, $CDCl_3$)

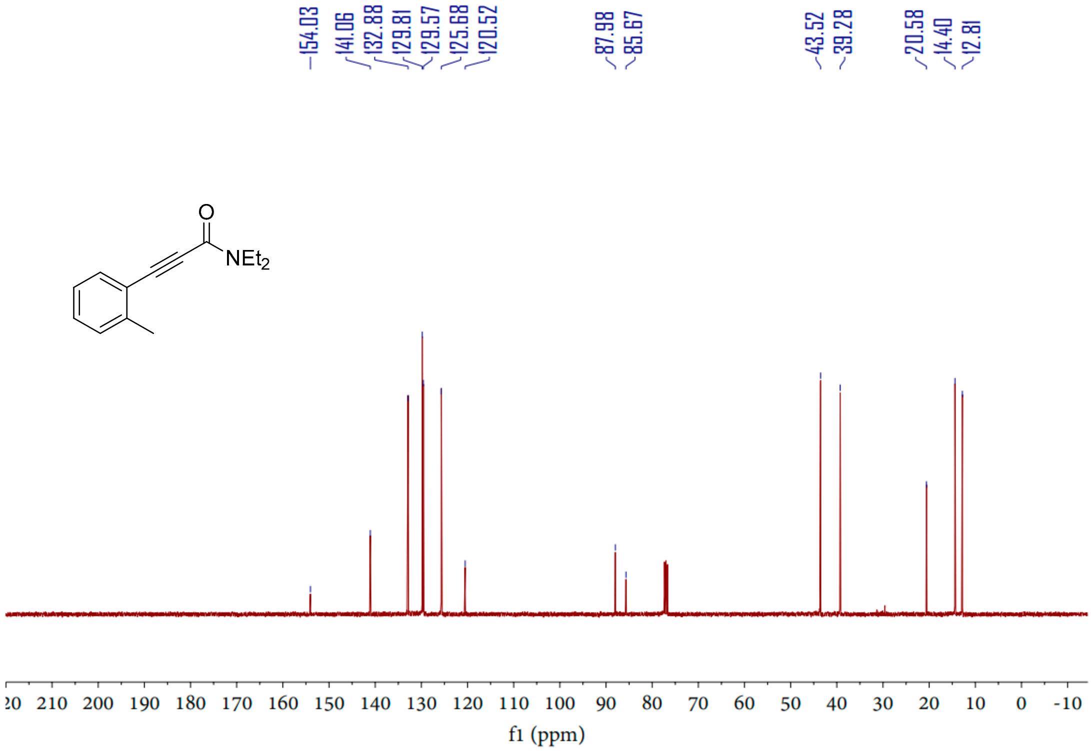

^{1}H NMR of **3j** in $CDCl_3$ (400 MHz, $CDCl_3$)

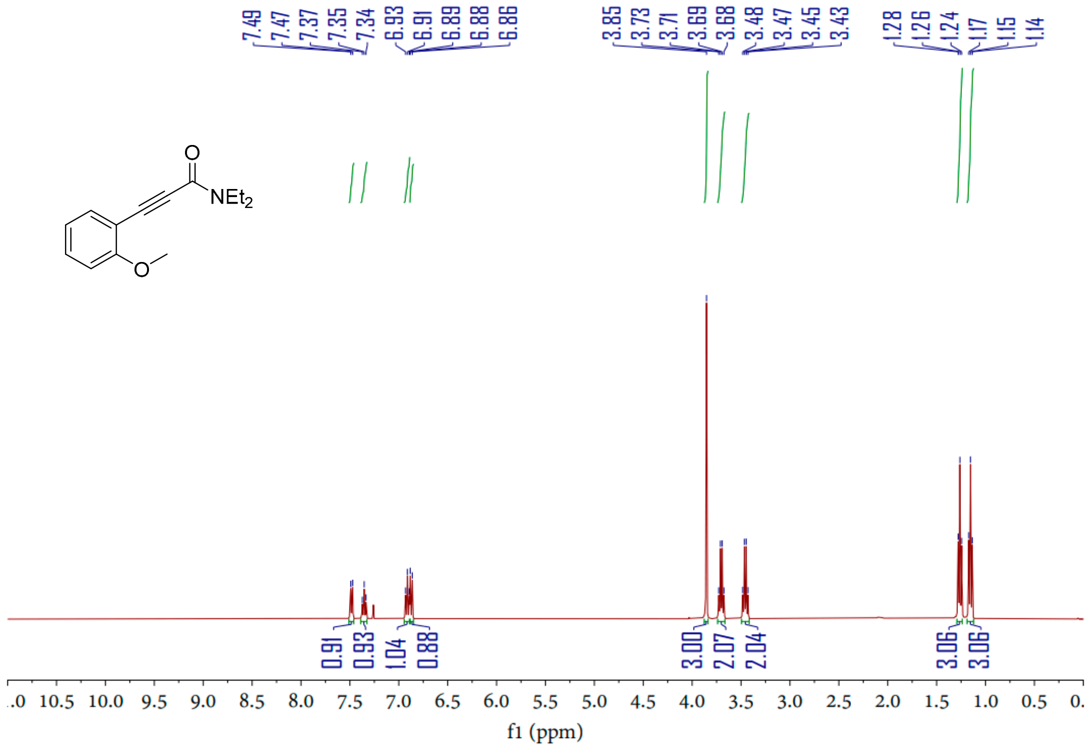

^{13}C NMR of **3j** in $CDCl_3$ (101 MHz, $CDCl_3$)

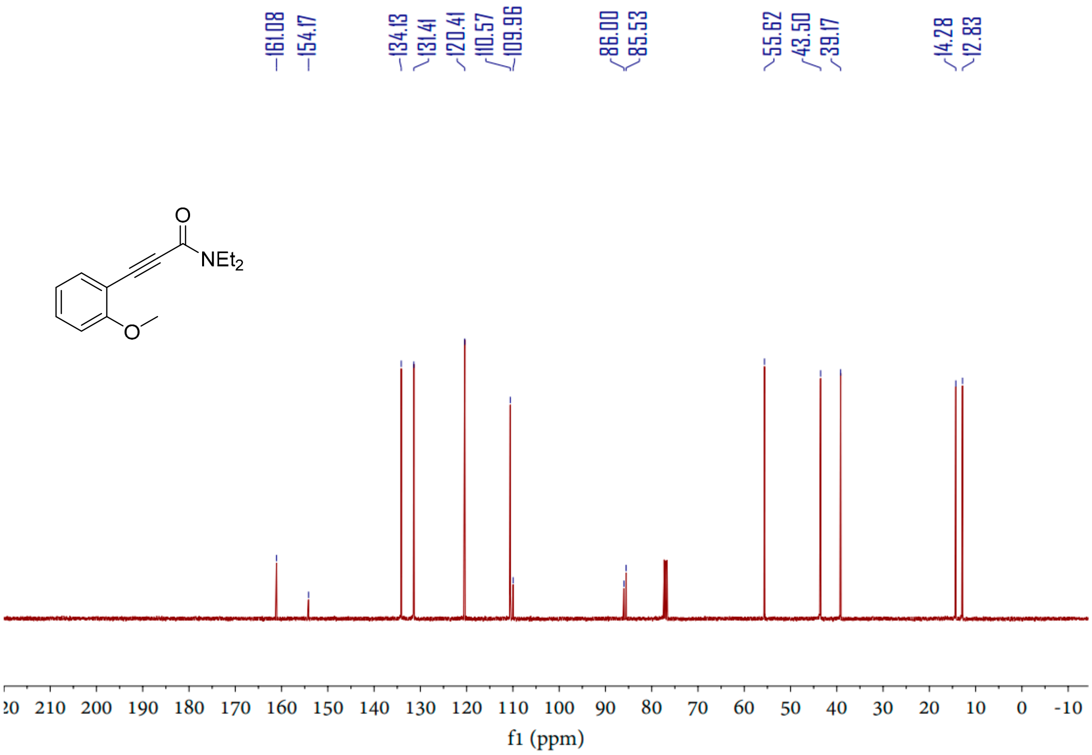

^{1}H NMR of **3k** in $CDCl_3$ (400 MHz, $CDCl_3$)

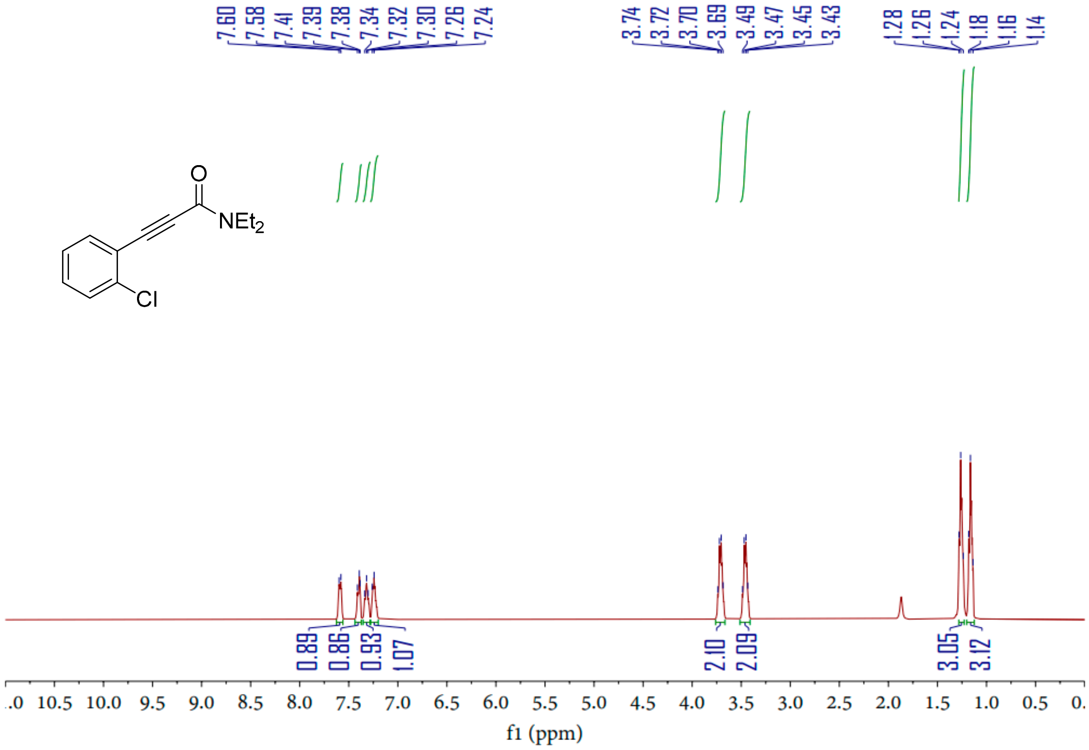

^{13}C NMR of **3k** in $CDCl_3$ (101 MHz, $CDCl_3$)

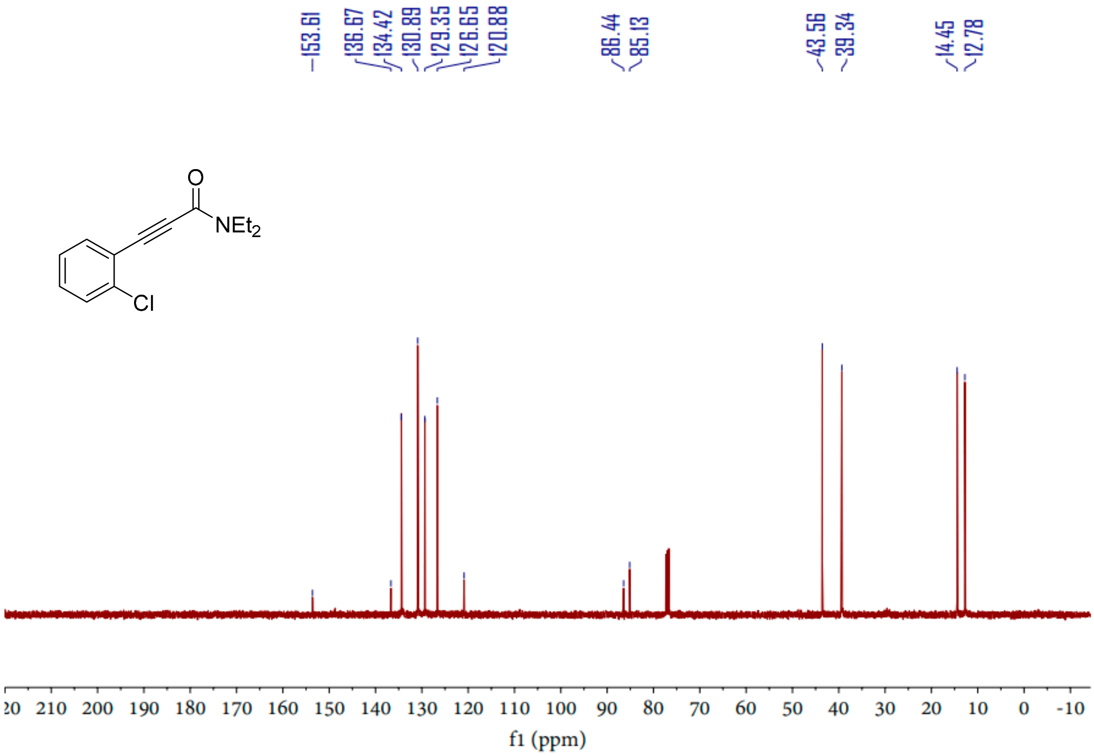

^{1}H NMR of **3l** in $CDCl_3$ (400 MHz, $CDCl_3$)

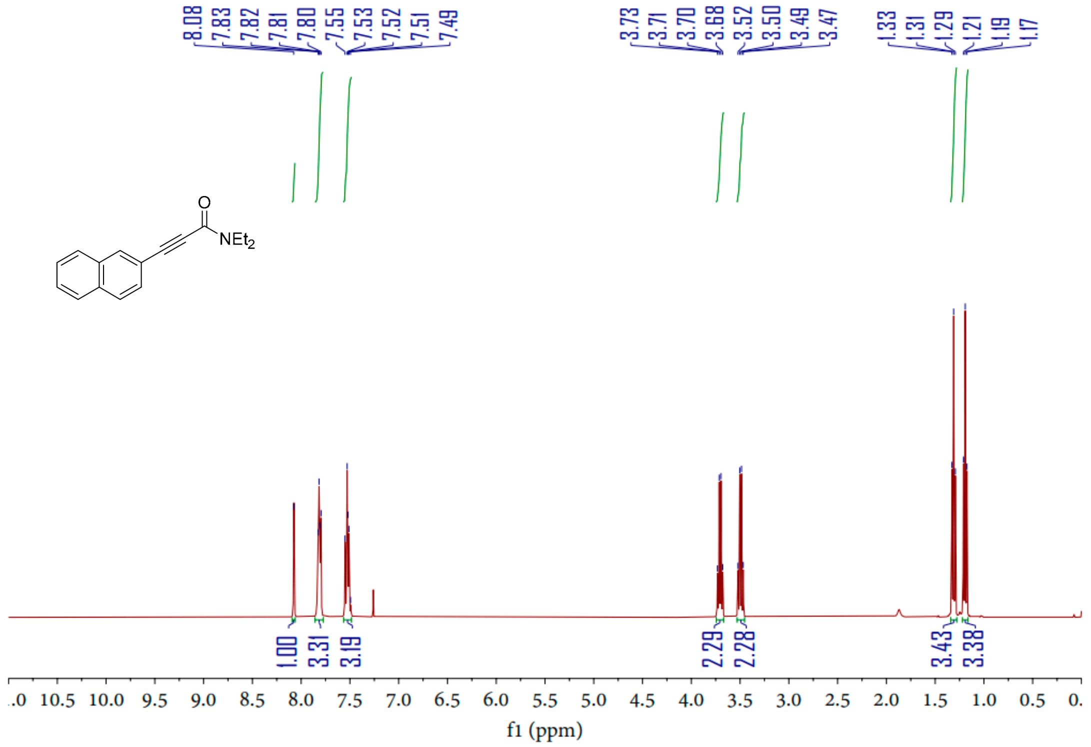

^{13}C NMR of **3l** in $CDCl_3$ (101 MHz, $CDCl_3$)

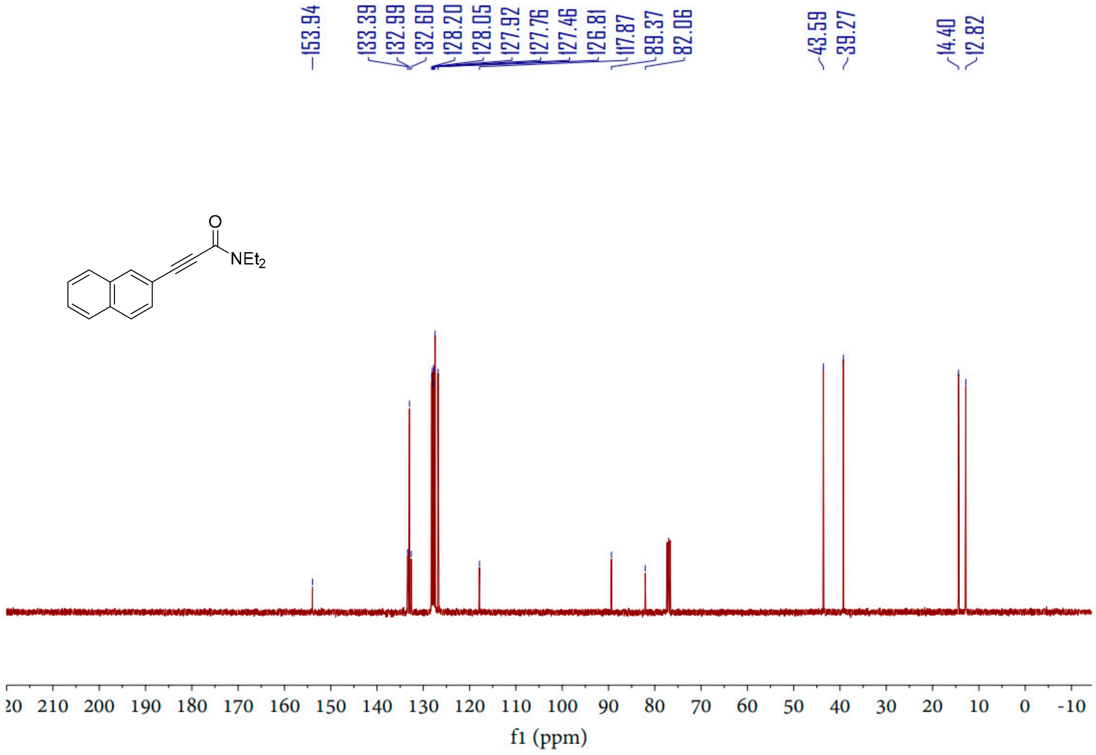

^{1}H NMR of **3m** in $CDCl_3$ (400 MHz, $CDCl_3$)

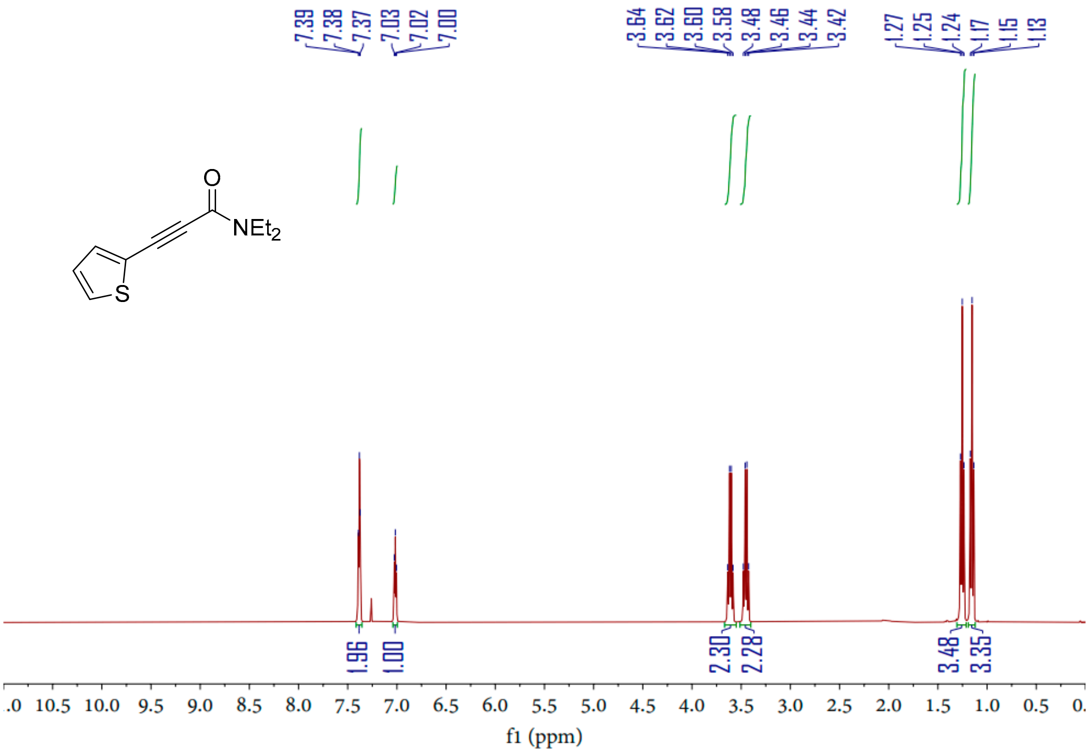

^{13}C NMR of **3m** in $CDCl_3$ (101 MHz, $CDCl_3$)

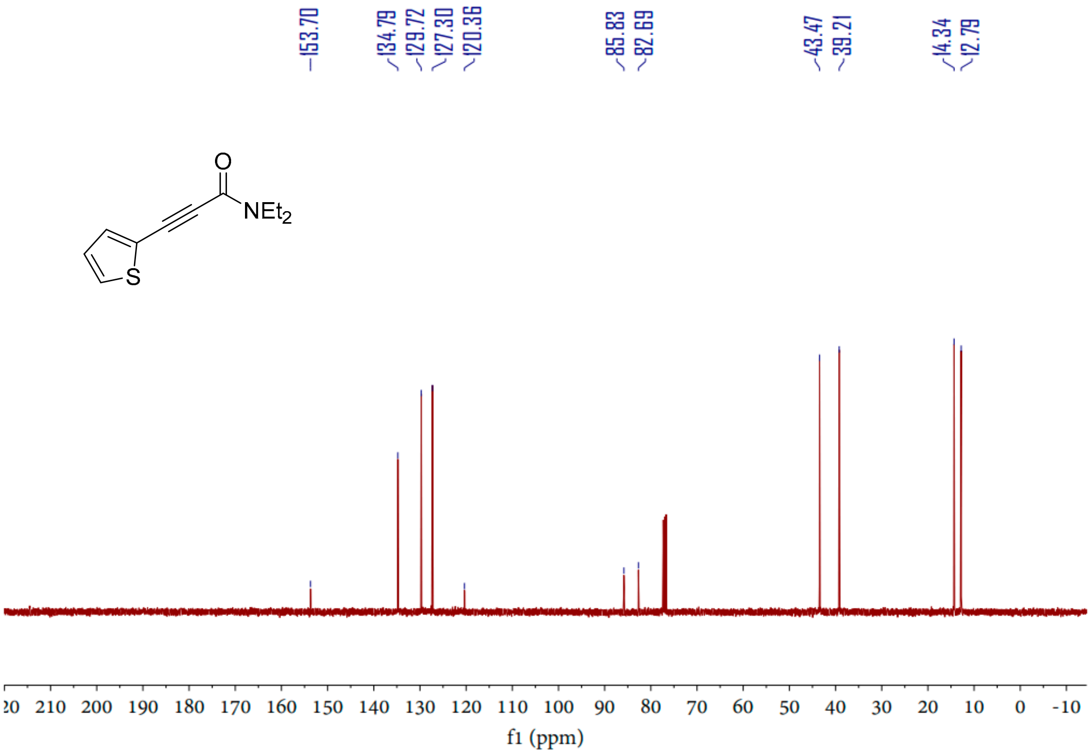

^{1}H NMR of **4a** in $CDCl_3$ (400 MHz, $CDCl_3$)

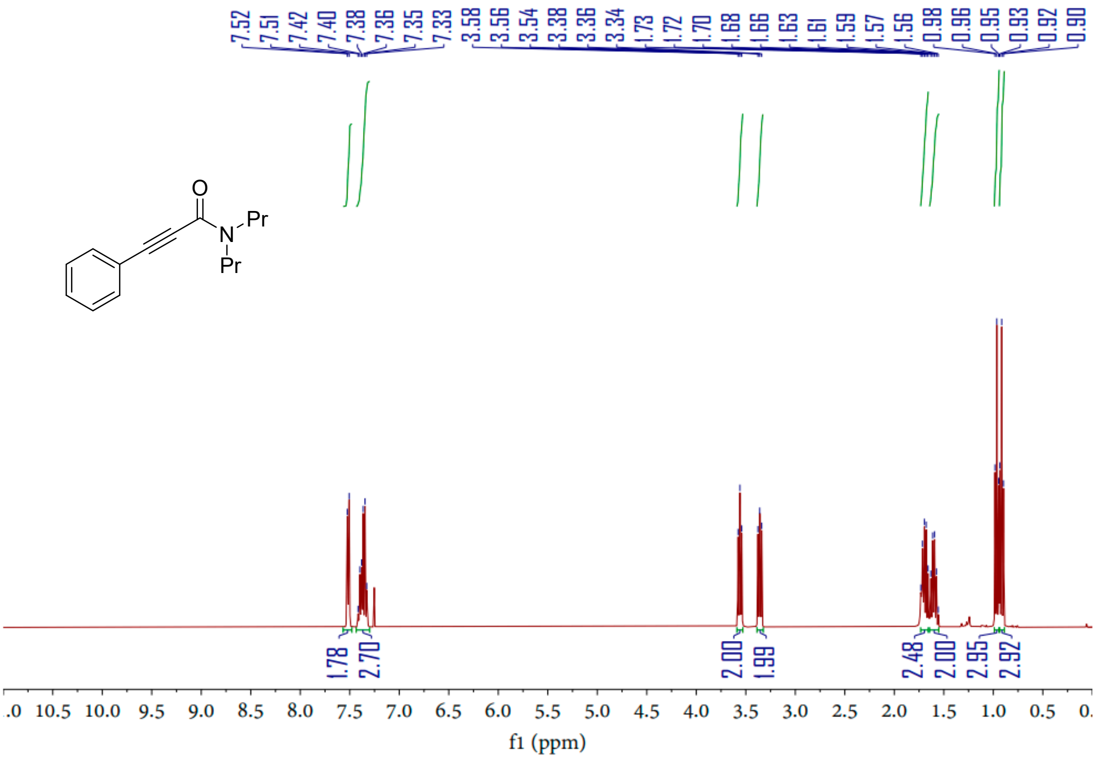

^{13}C NMR of **4a** in $CDCl_3$ (101 MHz, $CDCl_3$)

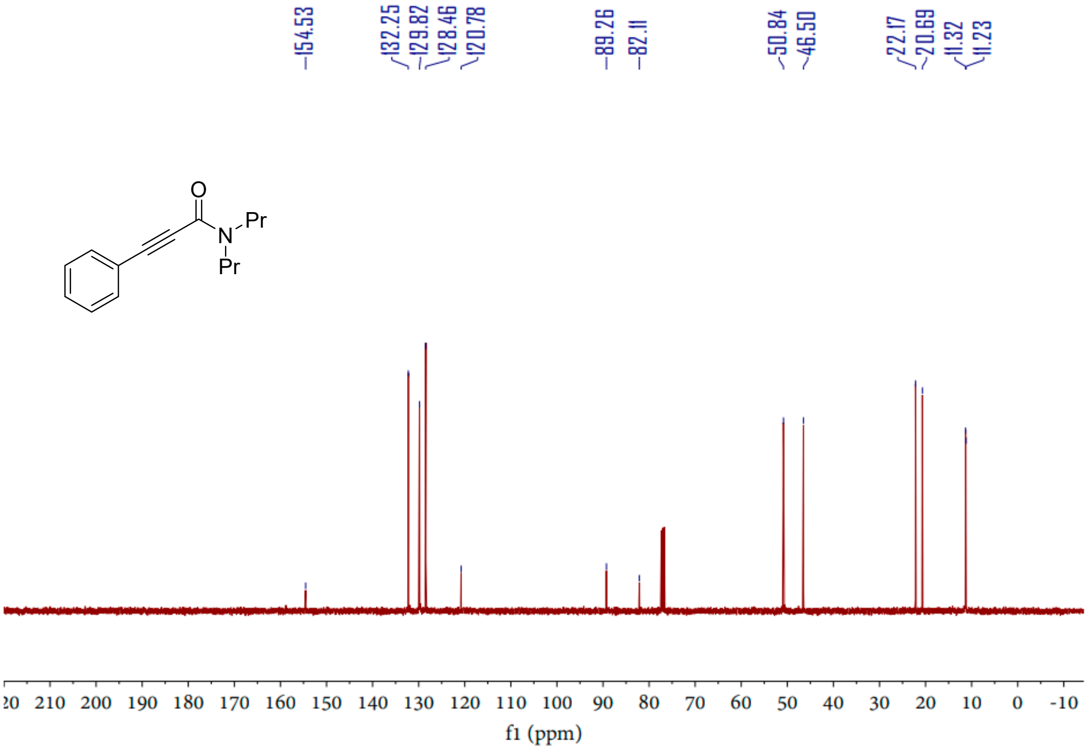

^{1}H NMR of **4b** in $CDCl_3$ (400 MHz, $CDCl_3$)

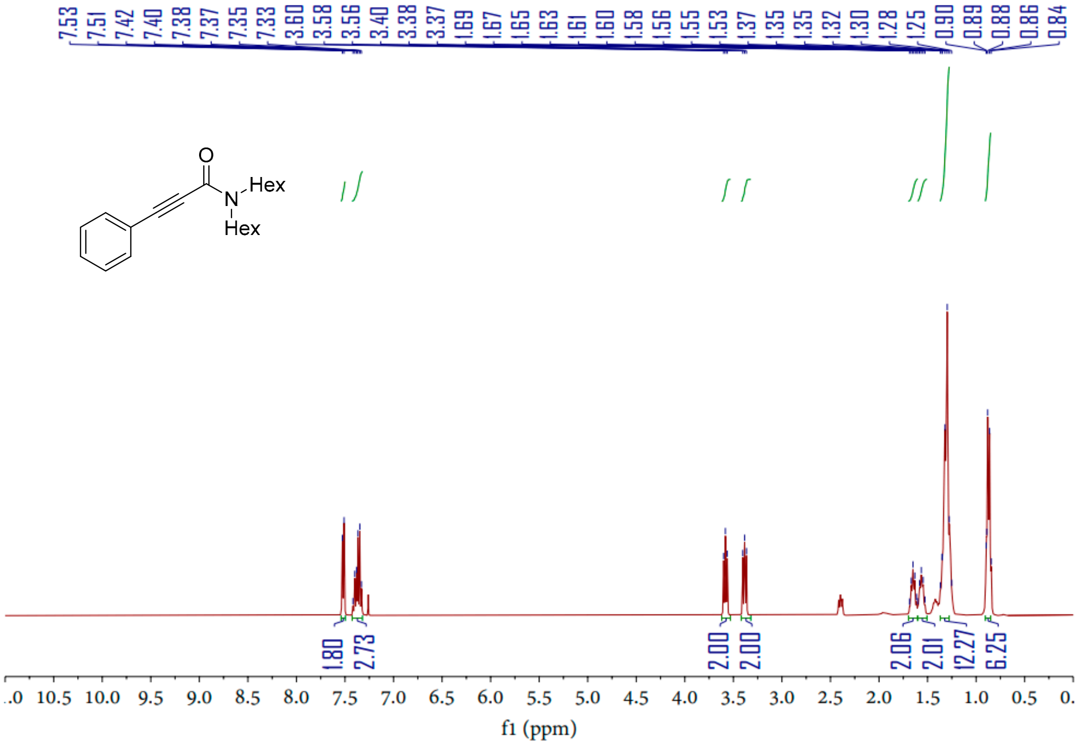

^{13}C NMR of **4b** in $CDCl_3$ (101 MHz, $CDCl_3$)

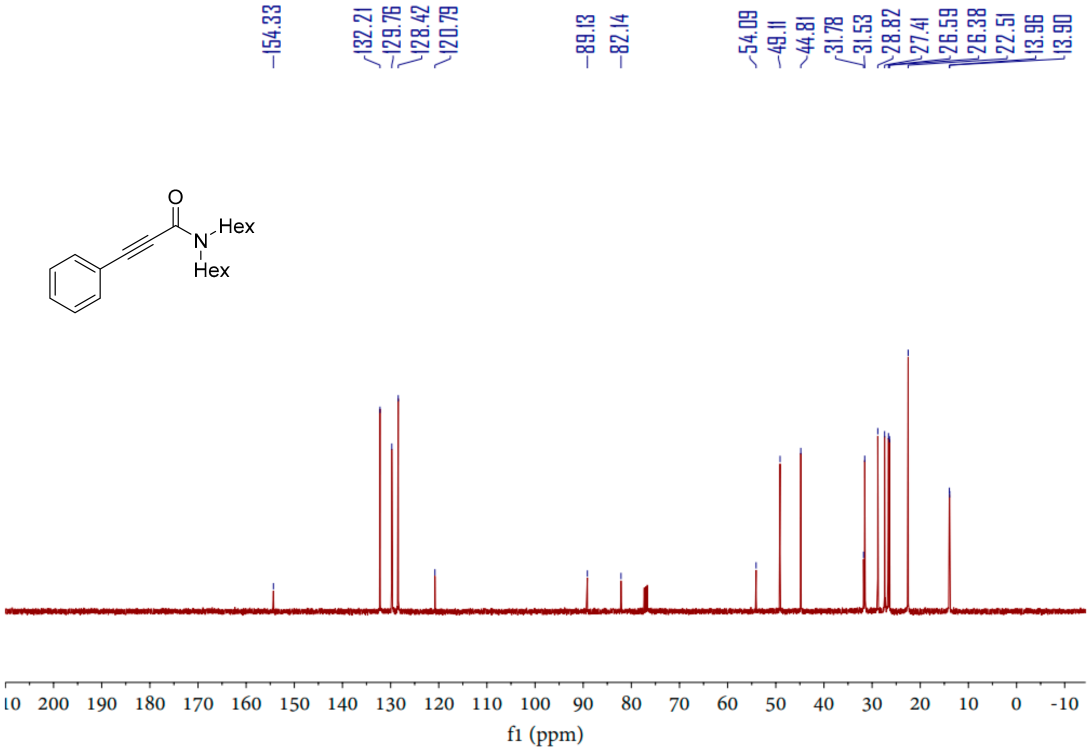

^{1}H NMR of **4c** in $CDCl_3$ (400 MHz, $CDCl_3$)

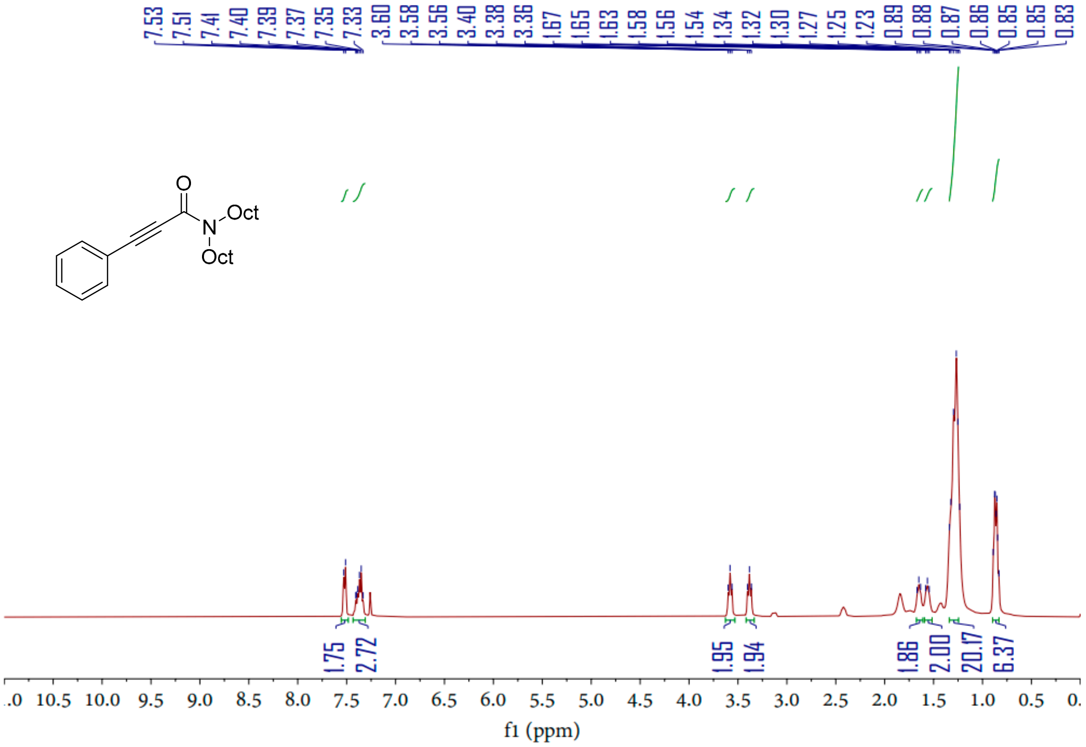

^{13}C NMR of **4c** in $CDCl_3$ (101 MHz, $CDCl_3$)

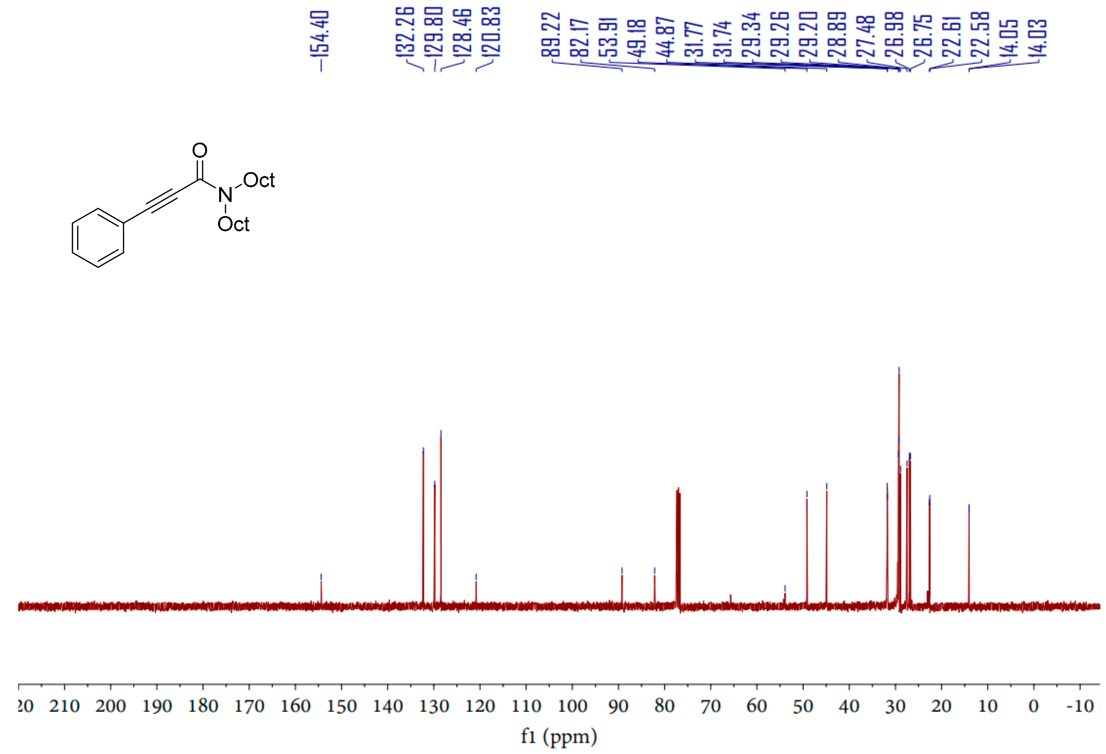

^{1}H NMR of **4d** in $CDCl_3$ (400 MHz, $CDCl_3$)

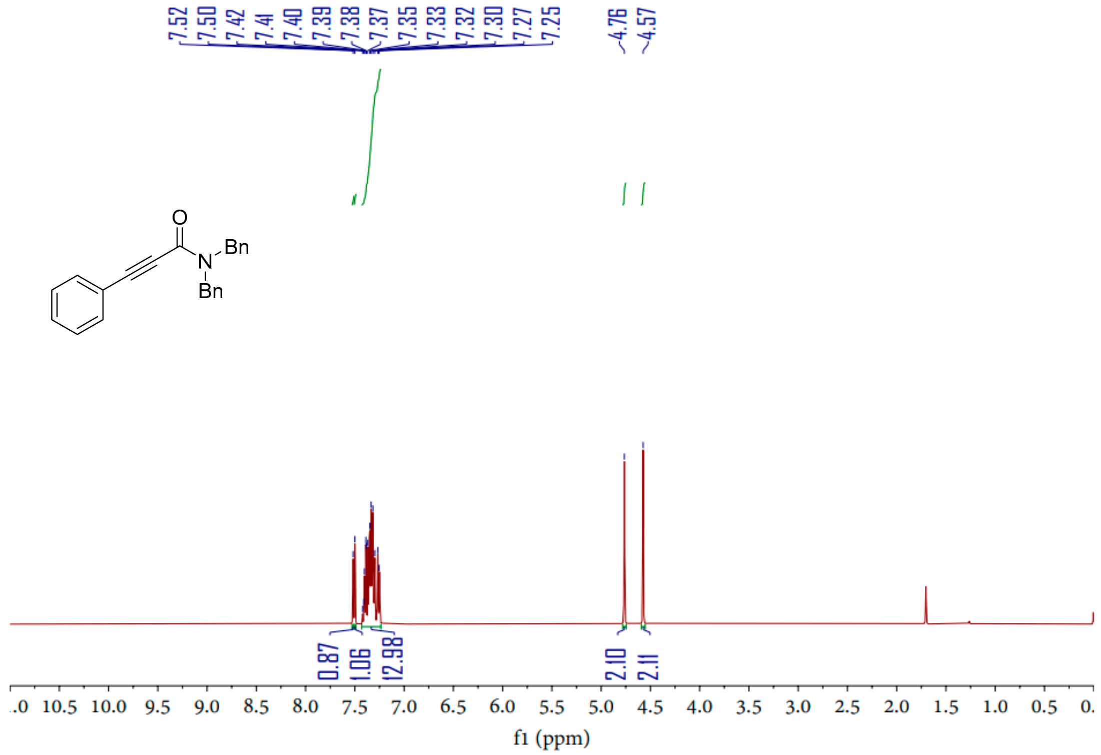

^{13}C NMR of **4d** in $CDCl_3$ (101 MHz, $CDCl_3$)

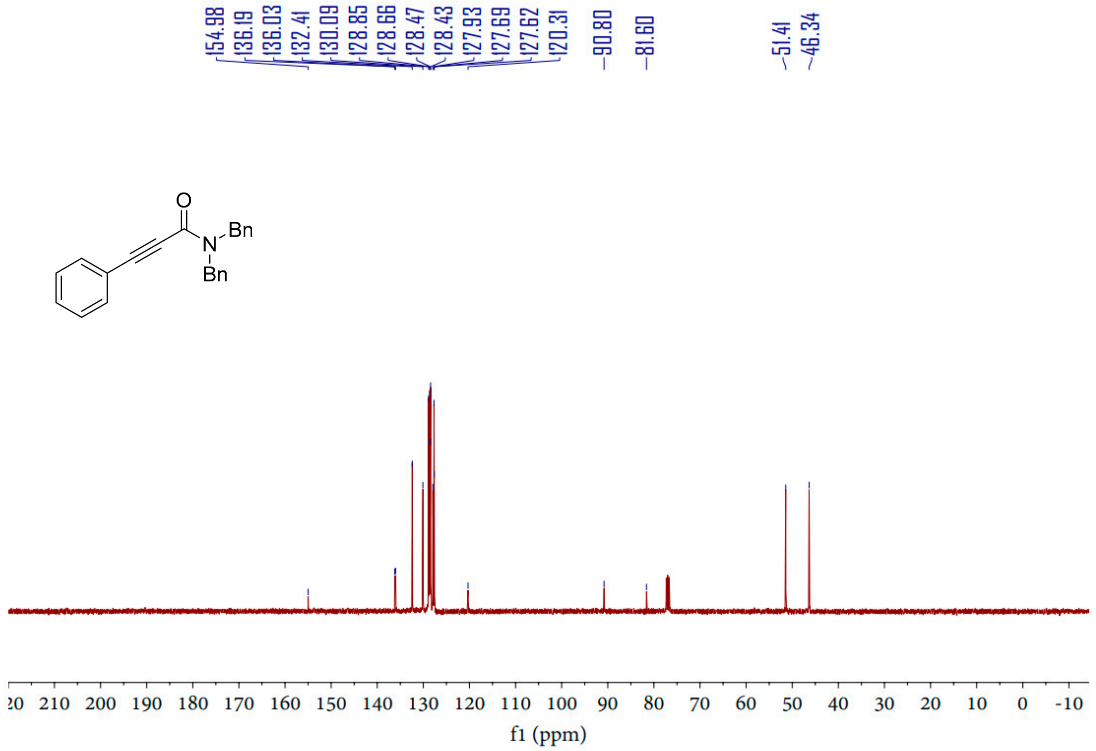

^{1}H NMR of **4e** in $CDCl_3$ (400 MHz, $CDCl_3$)

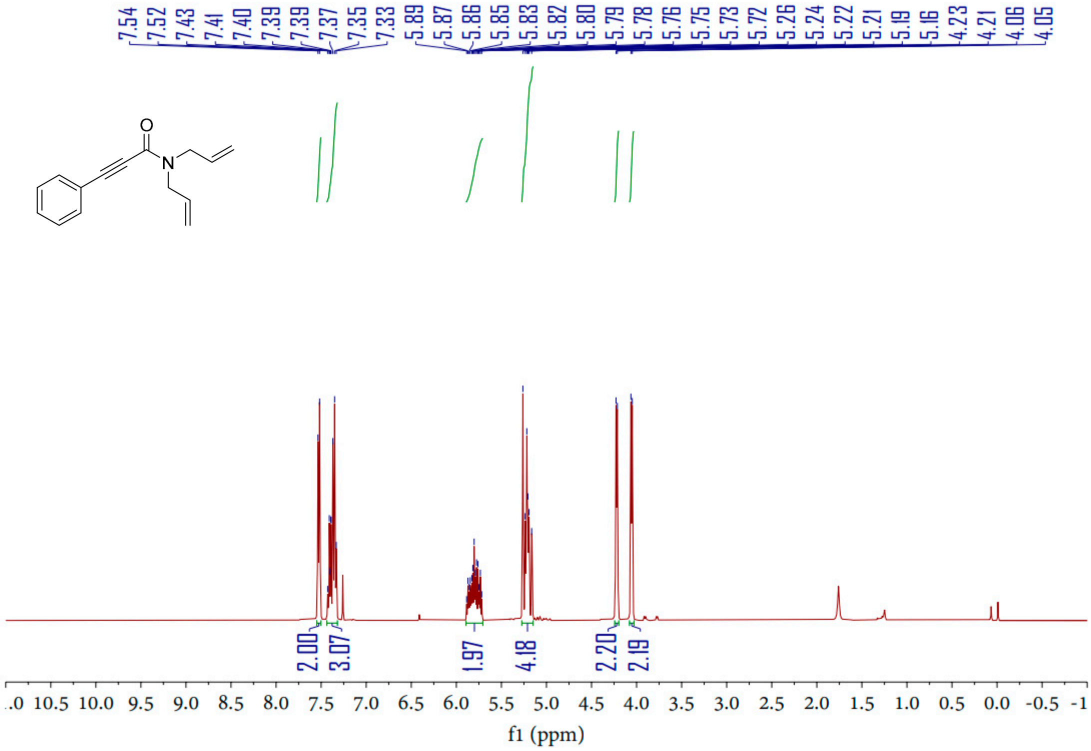

^{13}C NMR of **4e** in $CDCl_3$ (101 MHz, $CDCl_3$)

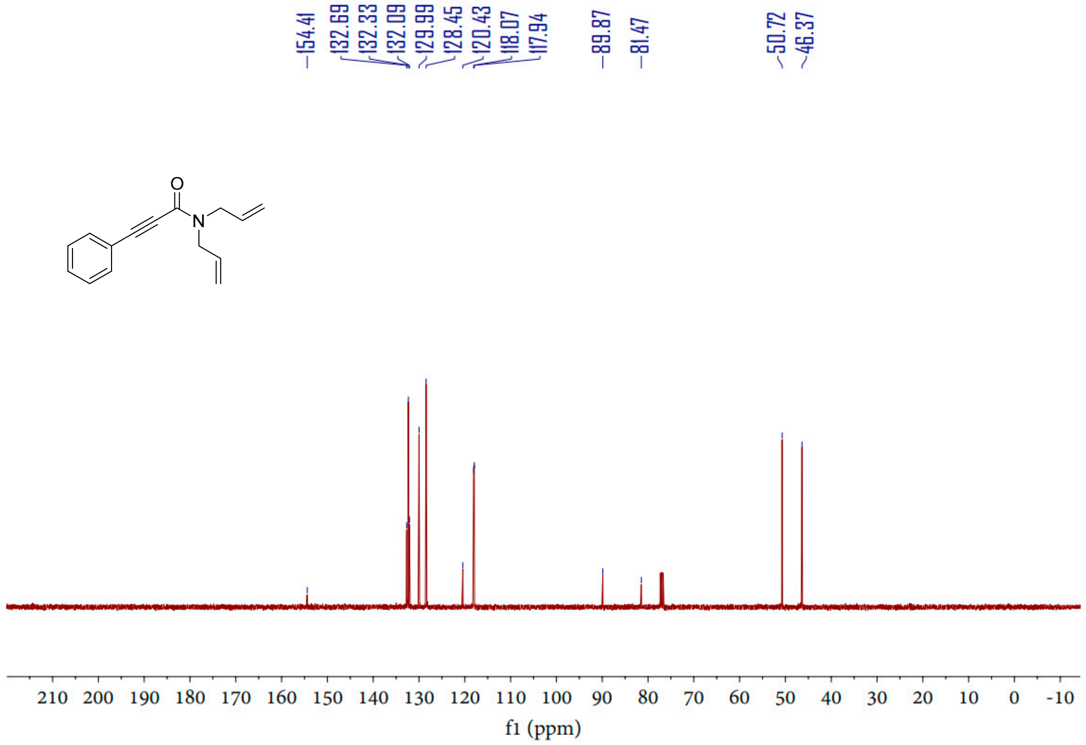

^{1}H NMR of **4f** in $CDCl_3$ (400 MHz, $CDCl_3$)

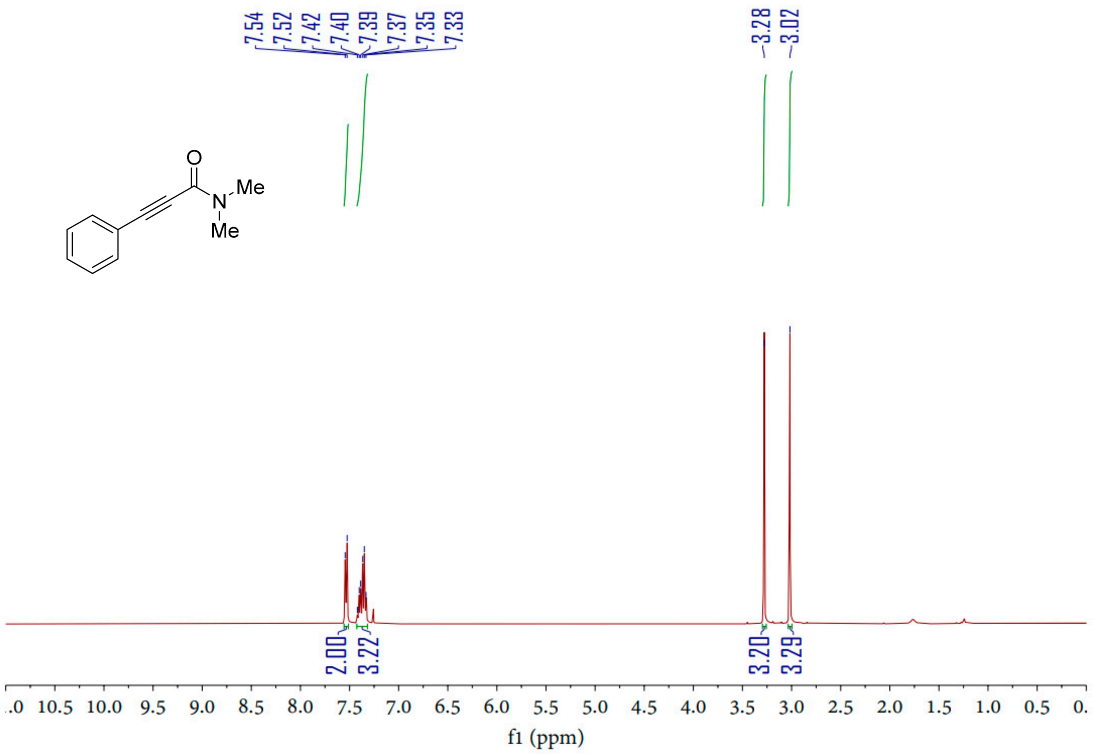

^{13}C NMR of **4f** in $CDCl_3$ (101 MHz, $CDCl_3$)

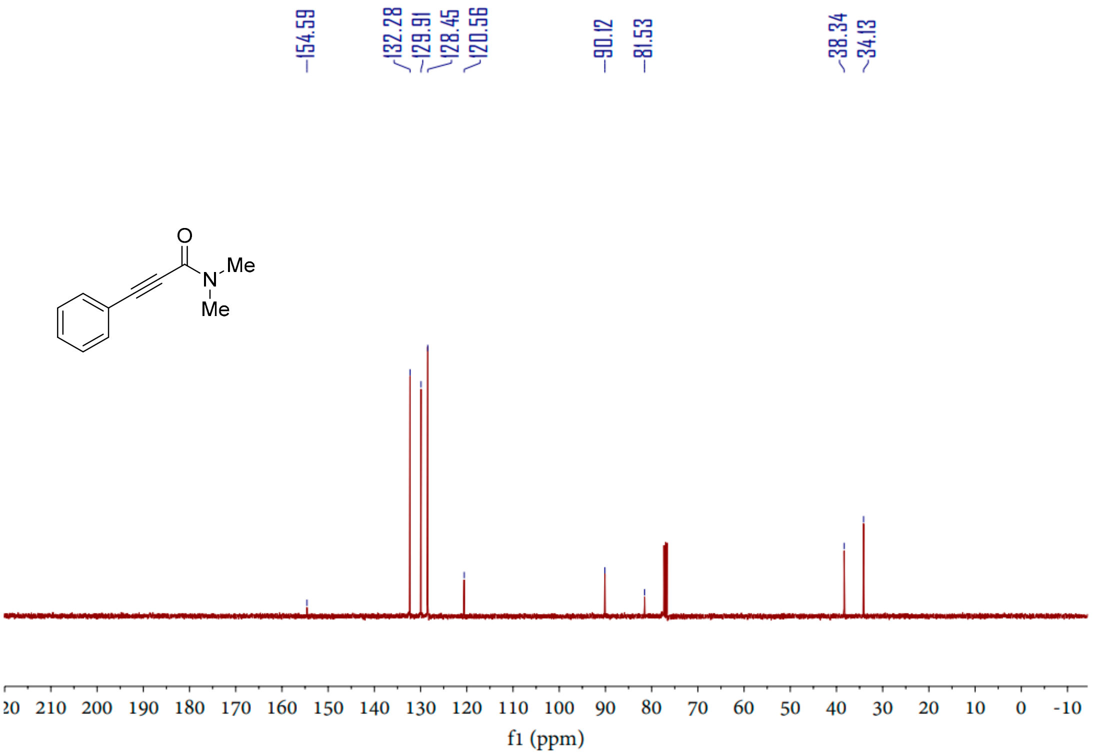

^{1}H NMR of **4h** in $CDCl_3$ (400 MHz, $CDCl_3$)

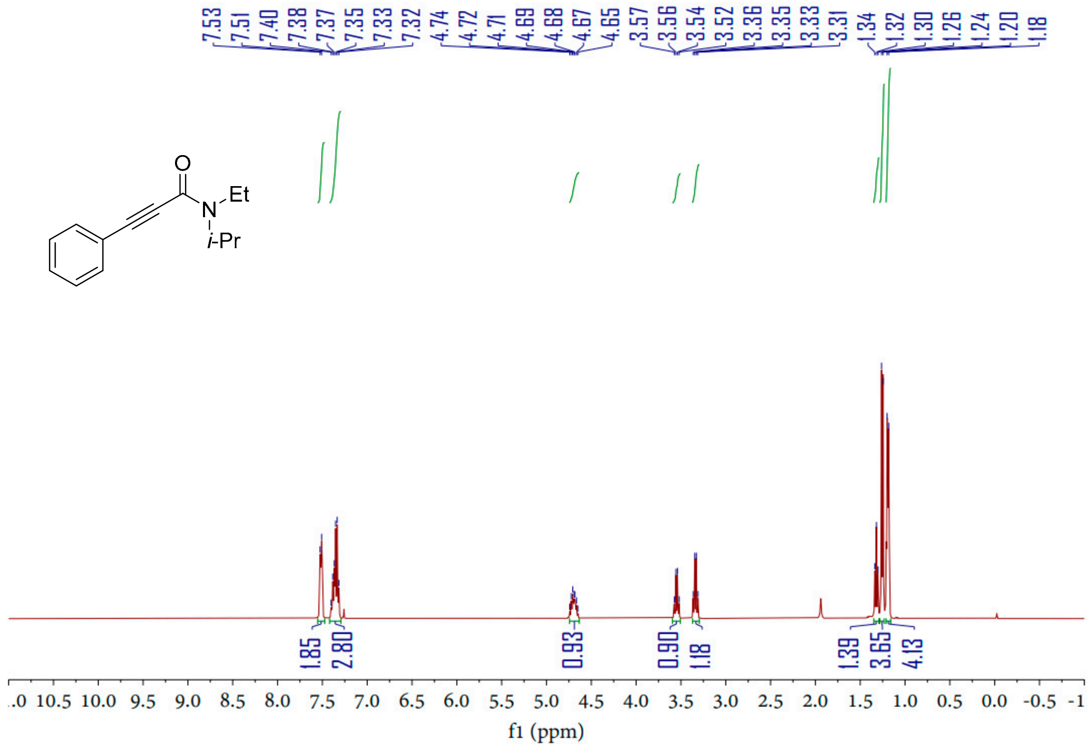

^{13}C NMR of **4h** in $CDCl_3$ (101 MHz, $CDCl_3$)

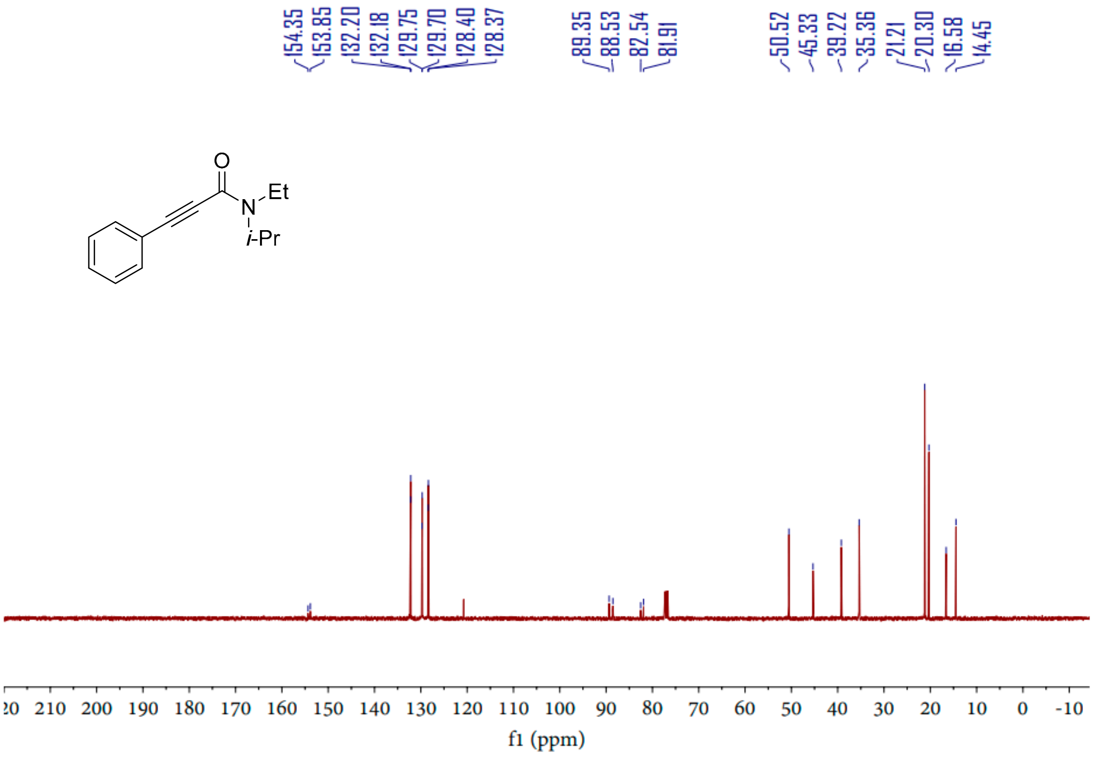

^{1}H NMR of **4i** in $CDCl_3$ (400 MHz, $CDCl_3$)

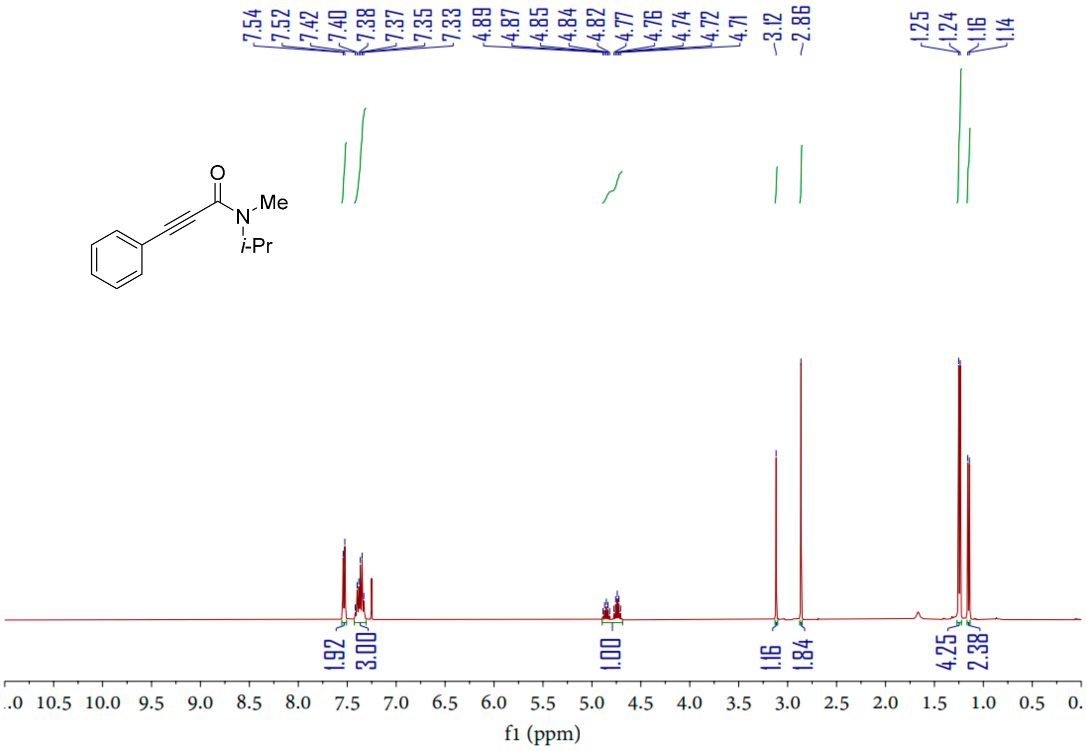

^{13}C NMR of **4i** in $CDCl_3$ (101 MHz, $CDCl_3$)

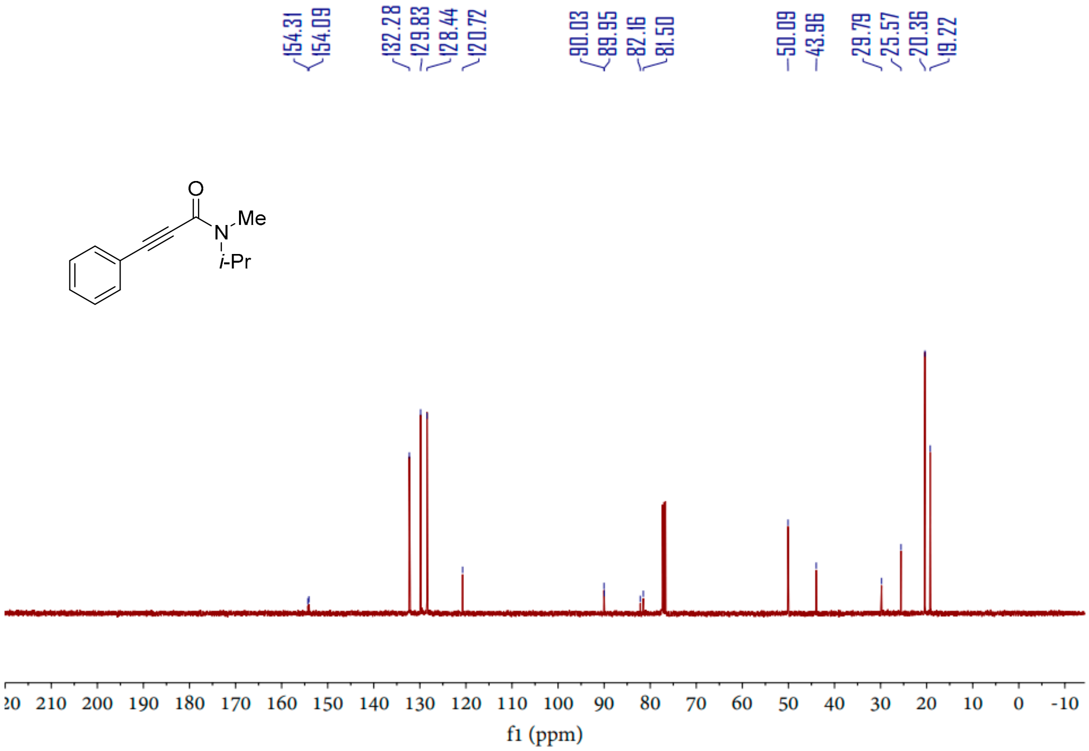

^{1}H NMR of **4k** in $CDCl_3$ (400 MHz, $CDCl_3$)

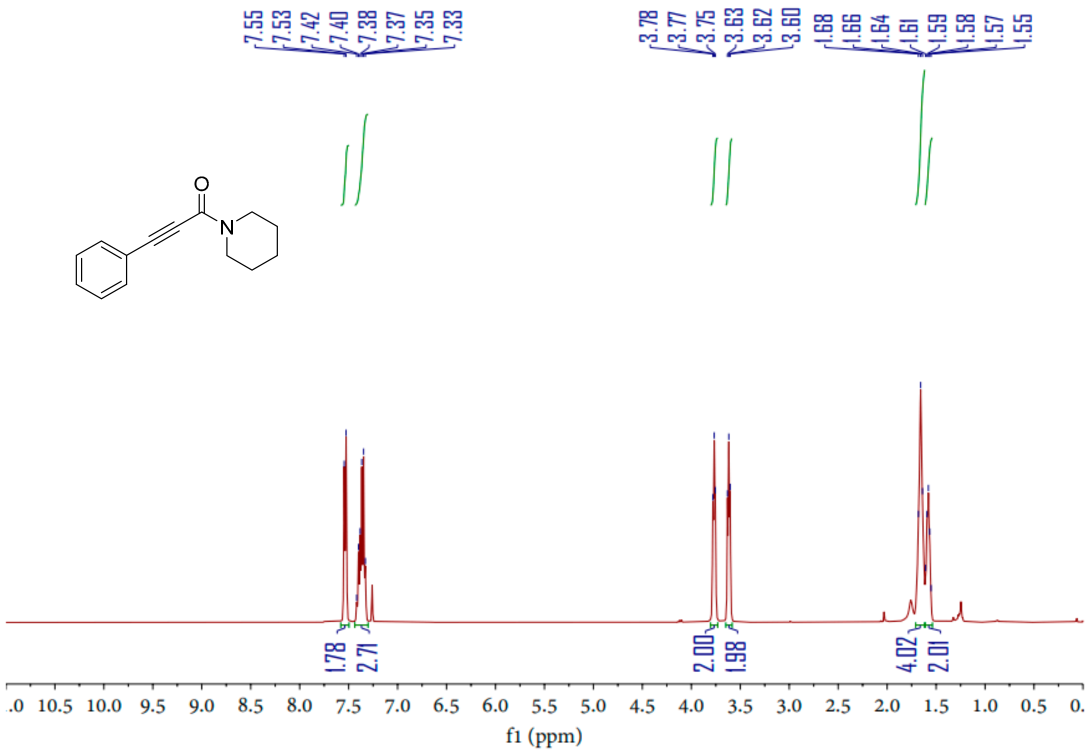

^{13}C NMR of **4k** in $CDCl_3$ (101 MHz, $CDCl_3$)

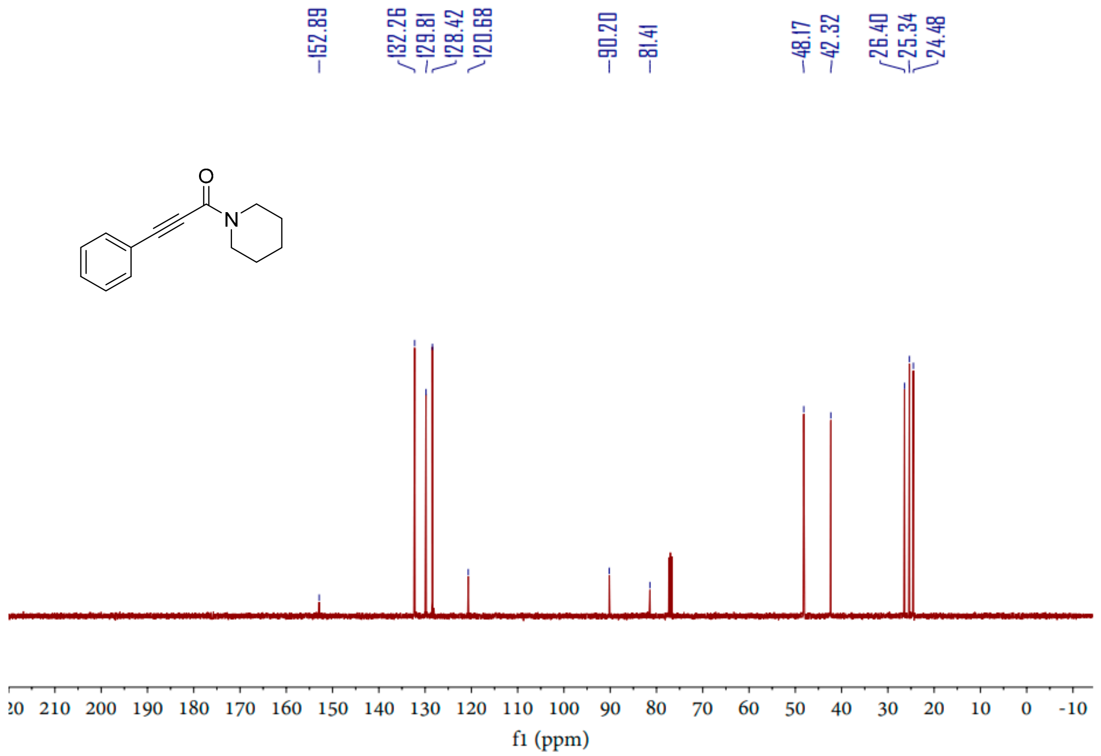

^{1}H NMR of **4l** in $CDCl_3$ (400 MHz, $CDCl_3$)

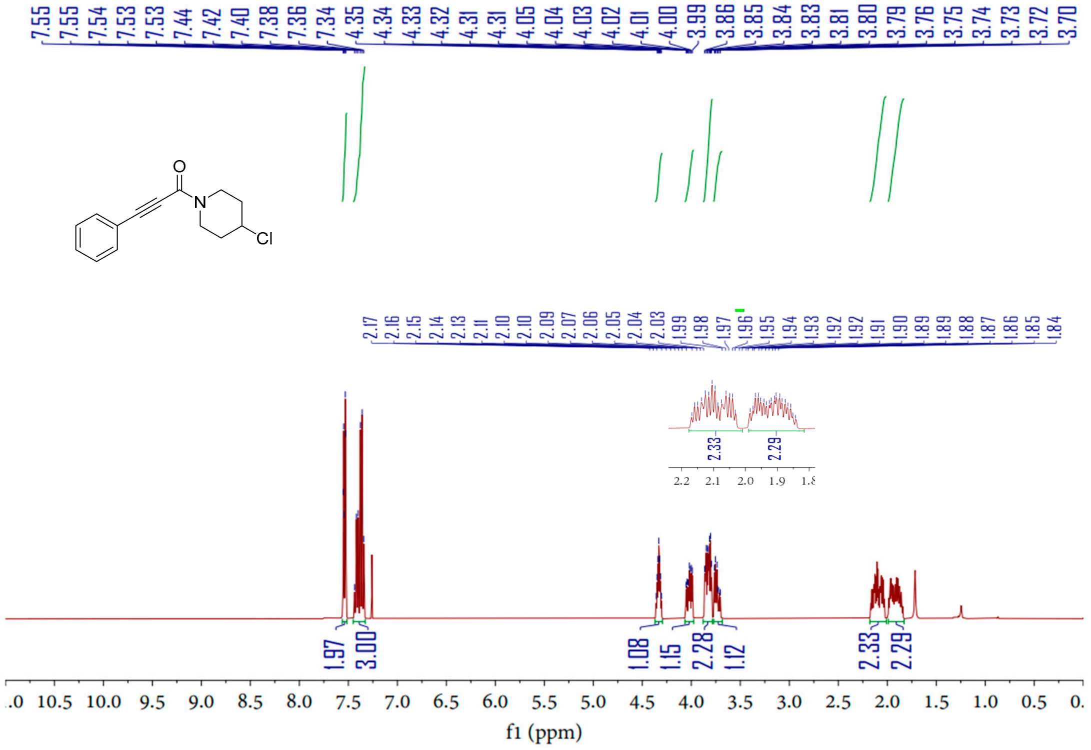

^{13}C NMR of **4l** in $CDCl_3$ (101 MHz, $CDCl_3$)

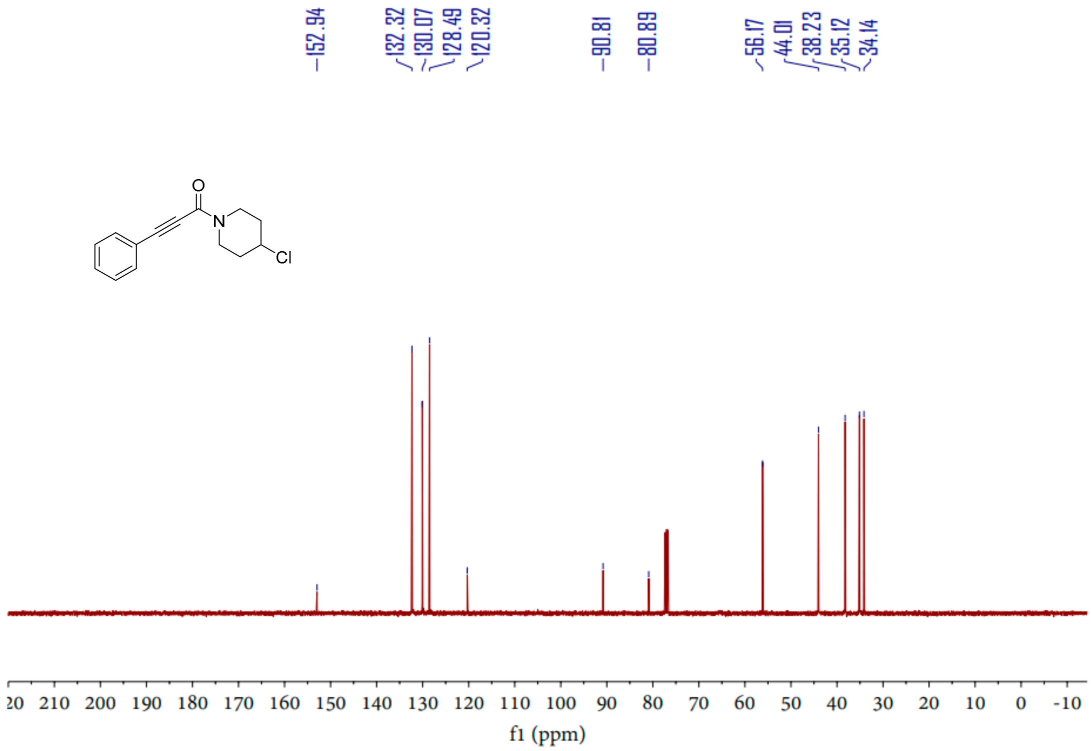

^{1}H NMR of **4m** in $CDCl_3$ (400 MHz, $CDCl_3$)

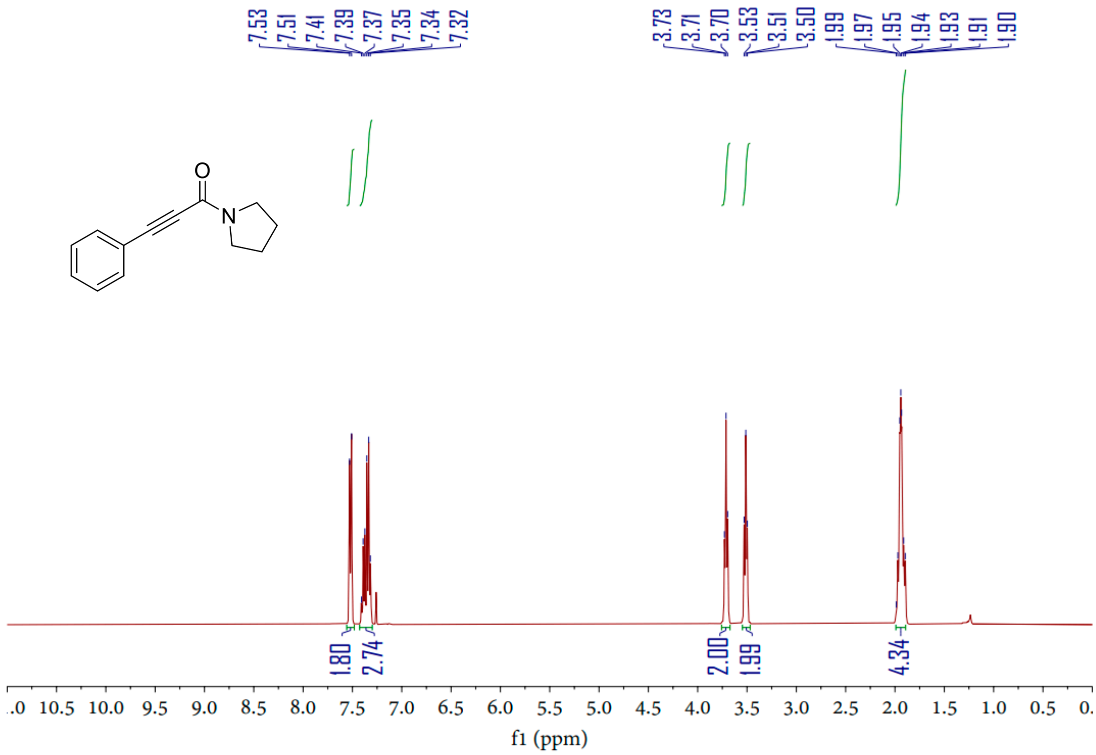

^{13}C NMR of **4m** in $CDCl_3$ (101 MHz, $CDCl_3$)

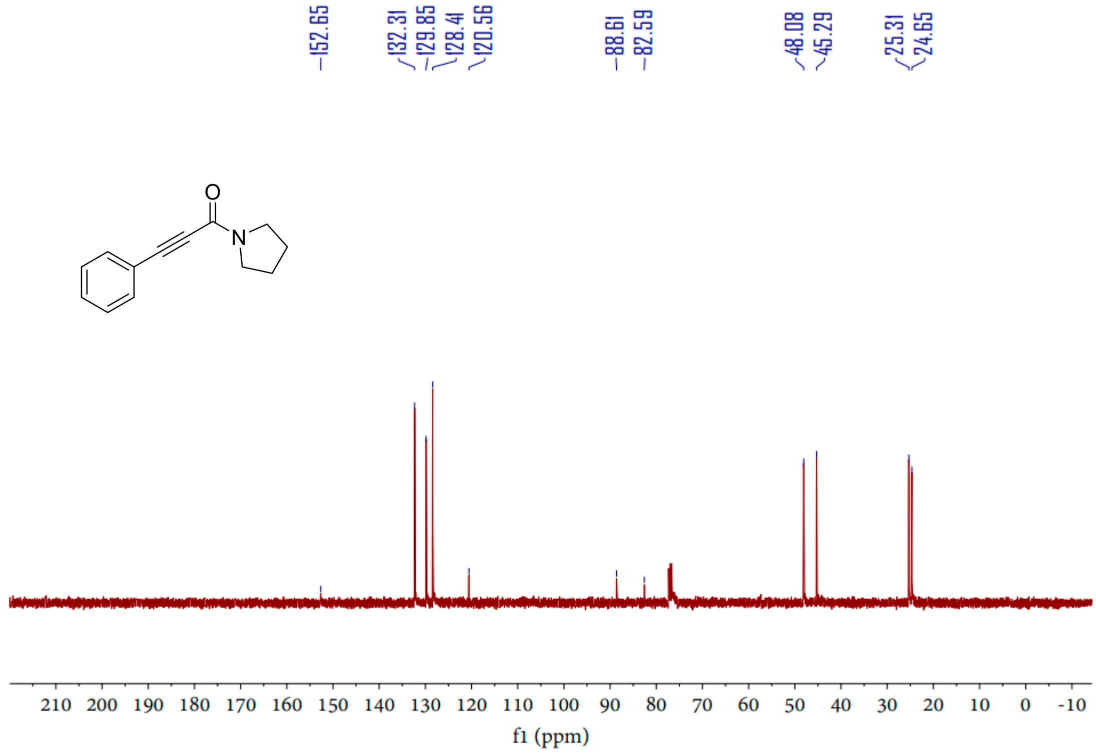

^{1}H NMR of **4n** in $CDCl_3$ (400 MHz, $CDCl_3$)

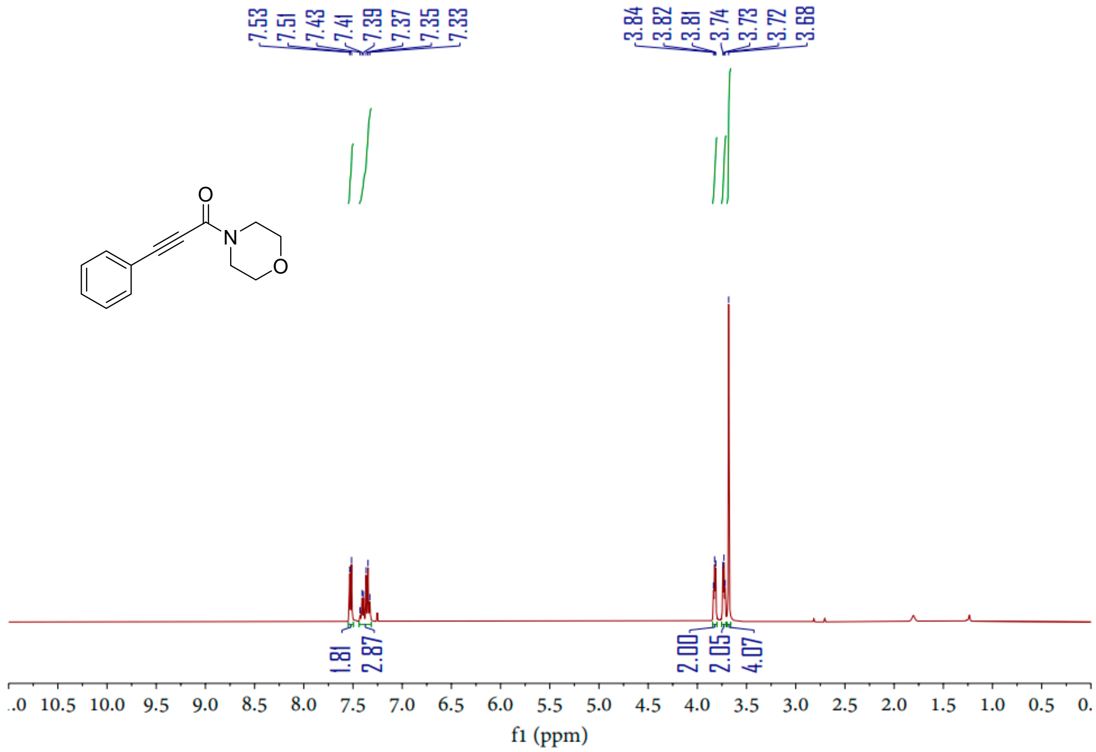

^{13}C NMR of **4n** in $CDCl_3$ (101 MHz, $CDCl_3$)

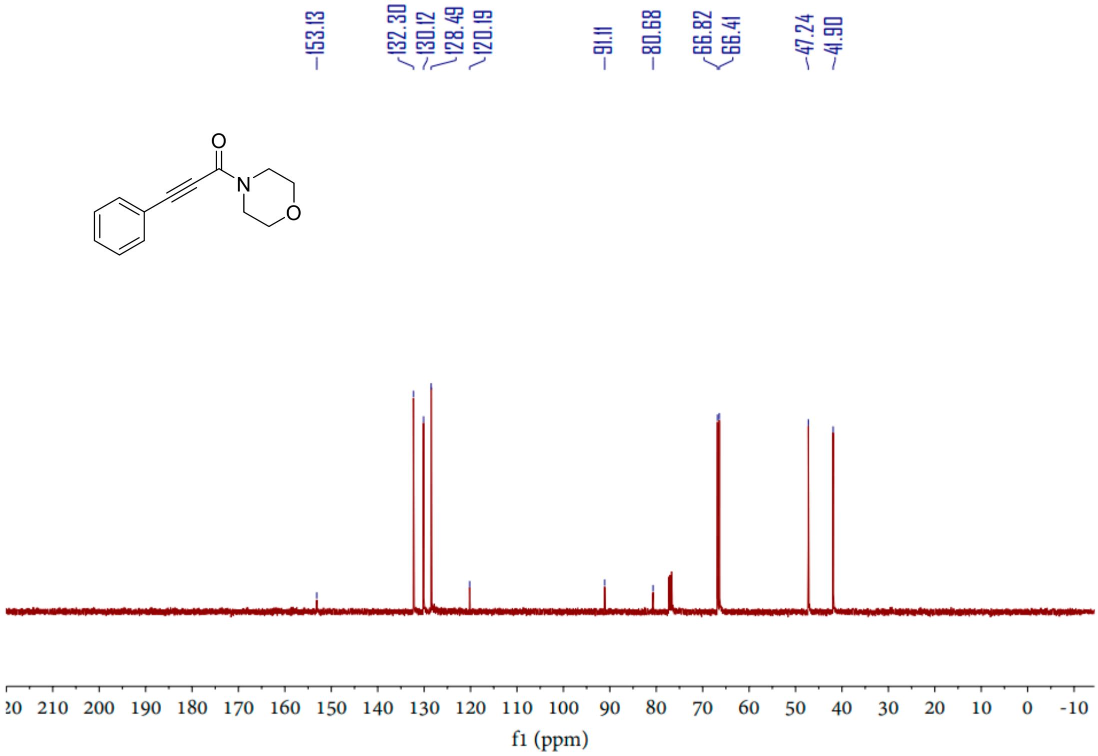

Supplement: Supplementary file 1 [file molecules-30-02955-s001.zip › molecules-3734216-supplementary.pdf]
